# Identification and characterization of stem cells in mammalian esophageal stratified squamous epithelia

**DOI:** 10.1093/jmcb/mjac038

**Published:** 2022-06-16

**Authors:** Yanan Yang, Guodong Deng, Lili Qiao, Hui Yuan, Xiaohong Yu, Lei Xu, Shih-Hsin Lu, Wei Jiang, Xiying Yu

**Affiliations:** Department of Etiology and Carcinogenesis, National Cancer Center/National Clinical Research Center for Cancer/Cancer Hospital, Chinese Academy of Medical Sciences and Peking Union Medical College, Beijing 100021, China; State Key Laboratory of Molecular Oncology, National Cancer Center/National Clinical Research Center for Cancer/Cancer Hospital, Chinese Academy of Medical Sciences and Peking Union Medical College, Beijing 100021, China; Department of Etiology and Carcinogenesis, National Cancer Center/National Clinical Research Center for Cancer/Cancer Hospital, Chinese Academy of Medical Sciences and Peking Union Medical College, Beijing 100021, China; State Key Laboratory of Molecular Oncology, National Cancer Center/National Clinical Research Center for Cancer/Cancer Hospital, Chinese Academy of Medical Sciences and Peking Union Medical College, Beijing 100021, China; Department of Etiology and Carcinogenesis, National Cancer Center/National Clinical Research Center for Cancer/Cancer Hospital, Chinese Academy of Medical Sciences and Peking Union Medical College, Beijing 100021, China; State Key Laboratory of Molecular Oncology, National Cancer Center/National Clinical Research Center for Cancer/Cancer Hospital, Chinese Academy of Medical Sciences and Peking Union Medical College, Beijing 100021, China; Department of Etiology and Carcinogenesis, National Cancer Center/National Clinical Research Center for Cancer/Cancer Hospital, Chinese Academy of Medical Sciences and Peking Union Medical College, Beijing 100021, China; State Key Laboratory of Molecular Oncology, National Cancer Center/National Clinical Research Center for Cancer/Cancer Hospital, Chinese Academy of Medical Sciences and Peking Union Medical College, Beijing 100021, China; Department of Etiology and Carcinogenesis, National Cancer Center/National Clinical Research Center for Cancer/Cancer Hospital, Chinese Academy of Medical Sciences and Peking Union Medical College, Beijing 100021, China; State Key Laboratory of Molecular Oncology, National Cancer Center/National Clinical Research Center for Cancer/Cancer Hospital, Chinese Academy of Medical Sciences and Peking Union Medical College, Beijing 100021, China; Department of Etiology and Carcinogenesis, National Cancer Center/National Clinical Research Center for Cancer/Cancer Hospital, Chinese Academy of Medical Sciences and Peking Union Medical College, Beijing 100021, China; State Key Laboratory of Molecular Oncology, National Cancer Center/National Clinical Research Center for Cancer/Cancer Hospital, Chinese Academy of Medical Sciences and Peking Union Medical College, Beijing 100021, China; Department of Etiology and Carcinogenesis, National Cancer Center/National Clinical Research Center for Cancer/Cancer Hospital, Chinese Academy of Medical Sciences and Peking Union Medical College, Beijing 100021, China; State Key Laboratory of Molecular Oncology, National Cancer Center/National Clinical Research Center for Cancer/Cancer Hospital, Chinese Academy of Medical Sciences and Peking Union Medical College, Beijing 100021, China; Beijing Key Laboratory for Carcinogenesis and Cancer Prevention, National Cancer Center/National Clinical Research Center for Cancer/Cancer Hospital, Chinese Academy of Medical Sciences and Peking Union Medical College, Beijing 100021, China; Department of Etiology and Carcinogenesis, National Cancer Center/National Clinical Research Center for Cancer/Cancer Hospital, Chinese Academy of Medical Sciences and Peking Union Medical College, Beijing 100021, China; State Key Laboratory of Molecular Oncology, National Cancer Center/National Clinical Research Center for Cancer/Cancer Hospital, Chinese Academy of Medical Sciences and Peking Union Medical College, Beijing 100021, China; Beijing Key Laboratory for Carcinogenesis and Cancer Prevention, National Cancer Center/National Clinical Research Center for Cancer/Cancer Hospital, Chinese Academy of Medical Sciences and Peking Union Medical College, Beijing 100021, China; Department of Etiology and Carcinogenesis, National Cancer Center/National Clinical Research Center for Cancer/Cancer Hospital, Chinese Academy of Medical Sciences and Peking Union Medical College, Beijing 100021, China; State Key Laboratory of Molecular Oncology, National Cancer Center/National Clinical Research Center for Cancer/Cancer Hospital, Chinese Academy of Medical Sciences and Peking Union Medical College, Beijing 100021, China; Beijing Key Laboratory for Carcinogenesis and Cancer Prevention, National Cancer Center/National Clinical Research Center for Cancer/Cancer Hospital, Chinese Academy of Medical Sciences and Peking Union Medical College, Beijing 100021, China

**Keywords:** esophageal stem cells, hemidesmosomes, Wnt signaling, homeostasis

## Abstract

Somatic stem cells are essential for the maintenance of tissue homeostasis. Despite its importance, how the esophageal stratified squamous epithelium executes its self-renewal and maintenance remains elusive. In this study, using 5-bromo-2′-deoxyuridine label-chase in rats *in vivo* and rat esophageal organoids *in vitro* together with genome-wide DNA methylation and single-cell RNA sequencing, we identified a slow-cycling/quiescent stem cell population that contained high levels of hemidesmosomes (HDs) and low levels of Wnt signaling localized spatially and randomly at the basal layer of the esophageal epithelium. Pseudotime cell trajectory analysis indicated that tissue cells originated from quiescent basal stem cells in the basal layer. Perturbations of HD component expression and/or Wnt signaling reduced the stem cell population in the basal layer of esophageal keratinocyte organoids, resulting in alterations in the organoid formation rate, size, morphogenesis, and proliferation–differentiation homeostasis. Furthermore, not only high levels of HDs and low levels of Wnt signaling but also an interplay between HD and Wnt signaling defined the stem cells of the basal layer. Hence, HDs and Wnt signaling are critical determinants for defining the stem cells of the basal layer required for tissue homeostasis in mammalian esophagi.

## Introduction

The endoderm-derived esophagus in mammals is an important organ of the digestive system between the hypopharynx and the stomach for transporting ingested foods. The mammalian esophagus originates from the anterior foregut, which also gives rise to the respiratory system during embryonic development. As these organs are specified via a process of respiratory–esophageal separation (RES), the esophageal epithelium forms a simple columnar epithelium and then transforms into a stratified multilayered squamous epithelium (Que, 2015; Zhang et al., 2017; Morrisey and Rustgi, 2018). The cellular and molecular mechanisms regulating RES and esophageal epithelial morphogenesis during embryonic development have been extensively studied in recent years (Jiang et al., 2015; Que, 2015; Zhang et al., 2018; Kim et al., 2019; Getachew et al., 2021). However, it is relatively unclear how the mature esophageal stratified squamous epithelium executes its self-renewal and maintenance of proliferation–differentiation homeostasis.

Adult stem cells are vital for tissue/organ maintenance. Two models, the homogeneity and heterogeneity models, have been proposed for self-renewal and maintenance of the tissue homeostasis of the mature stratified squamous esophageal epithelium (Zhang et al., 2017, 2021; Lin et al., 2018). The homogeneity model hypothesizes that cells in the basal layer consist of one single population that can function as stem-like progenitors via the cell division cycle to produce daughter cells. Thus, these cells are randomly chosen to remain as progenitors or differentiate into suprabasal cells (SPBCs) for further differentiation (Marques-Pereira and Leblond, 1965; Doupe et al., 2012; Alcolea et al., 2014; Piedrafita et al., 2020). In contrast, the heterogeneity model offers an alternative possibility in which, like other organs such as the colon and the stomach with a simple columnar epithelium or the skin with a stratified squamous epithelium, the basal layers of mammalian esophagi have a slow-cycling or quiescent stem cell subpopulation that can be self-renewing, giving rise to fast-dividing progenitor cells in the basal layers and/or all other differentiated lineages in the suprabasal layers and the differentiated layers (Zhang et al., 2021). In support of this model, asymmetrical cell division and cells with specific stemness-related markers have been found in the basal layers of mammalian esophagi (Seery and Watt, 2000; Okumura et al., 2003; Croagh et al., 2007; Pan et al., 2013; Barbera et al., 2015). Tissue reconstitution and organoid formation have indicated that cells isolated with various stemness-related markers from mammalian esophagi can efficiently regenerate a completely stratified multilayered squamous epithelium when compared with cells without these markers (Kalabis et al., 2008; DeWard et al., 2014; Jeong et al., 2016; Giroux et al., 2017). As single-cell RNA sequencing (scRNA-seq) enables identification of cell populations at single-cell resolution, Busslinger et al. (2021) recently identified a quiescent *Col17a1^high^KRT15^high^* stem/progenitor cell population from the basal cell layer of human esophagi by scRNA-seq. Hence, an accumulation of evidence supports the heterogeneity of the basal cells of mammalian esophagi. However, it remains unclear what proportion(s) of basal cells are stem cells, where the stem cells are located, and how the stem cells are defined to maintain proliferation–differentiation homeostasis in the esophageal stratified squamous epithelium. In this study, we report the identification and characterization of stem cells in mammalian esophagi.

## Results

### Identification of slow-cycling/quiescent basal cells in mammalian esophagi

We sought to determine whether a stem cell subpopulation could be detected in the basal layers of mammalian esophagi. Esophageal tissues from rats, mice, and humans were collected, fixed, and stained with hematoxylin–eosin (H&E) (Supplementary Figure S1A) and various cell markers (Supplementary Figure S1B–D). While H&E staining revealed the typical stratified squamous epithelia in rat, mouse, and human esophageal tissues, the undifferentiated keratinocyte marker cytokeratin14 (CK14) marked cells only in the basal layer(s), and the differentiated keratinocyte marker cytokeratin13 (CK13) marked cells only in the suprabasal layer(s) and the differentiated layer(s). The stemness-related marker ΔNp63 (simply called p63) was detected in the basal cells but not SPBCs in these tissues, whereas other stemness-related markers, SOX2, BMI1, and OCT4, and the DNA synthesis marker PCNA were stained in both basal cells and SPBCs. Previously, the neurotrophin receptor component P75NTR and hemidesmosome (HD) components integrin α6 (ITGα6) and β4 (ITGβ4) have been indicated as potential stem cell markers of esophageal keratinocytes (Okumura et al., 2003; DeWard et al., 2014). Immunofluorescence staining showed that P75NTR, ITGα6, and ITGβ4 were clearly detectable at the cell membranes of the basal cells (Supplementary Figure S1B and D). While P75NTR was stained ubiquitously, ITGα6 and ITGβ4 staining showed subtle variations in the basement membranes of the basal cells (Supplementary Figure S1B and D). Quantification of the staining intensities of ITGα6 and ITGβ4 was performed, but the results were inconclusive. In summary, these results indicated that these markers could distinguish basal cells and/or SPBCs but could not precisely mark stem cells in mammalian esophagi.

**Figure 1:**
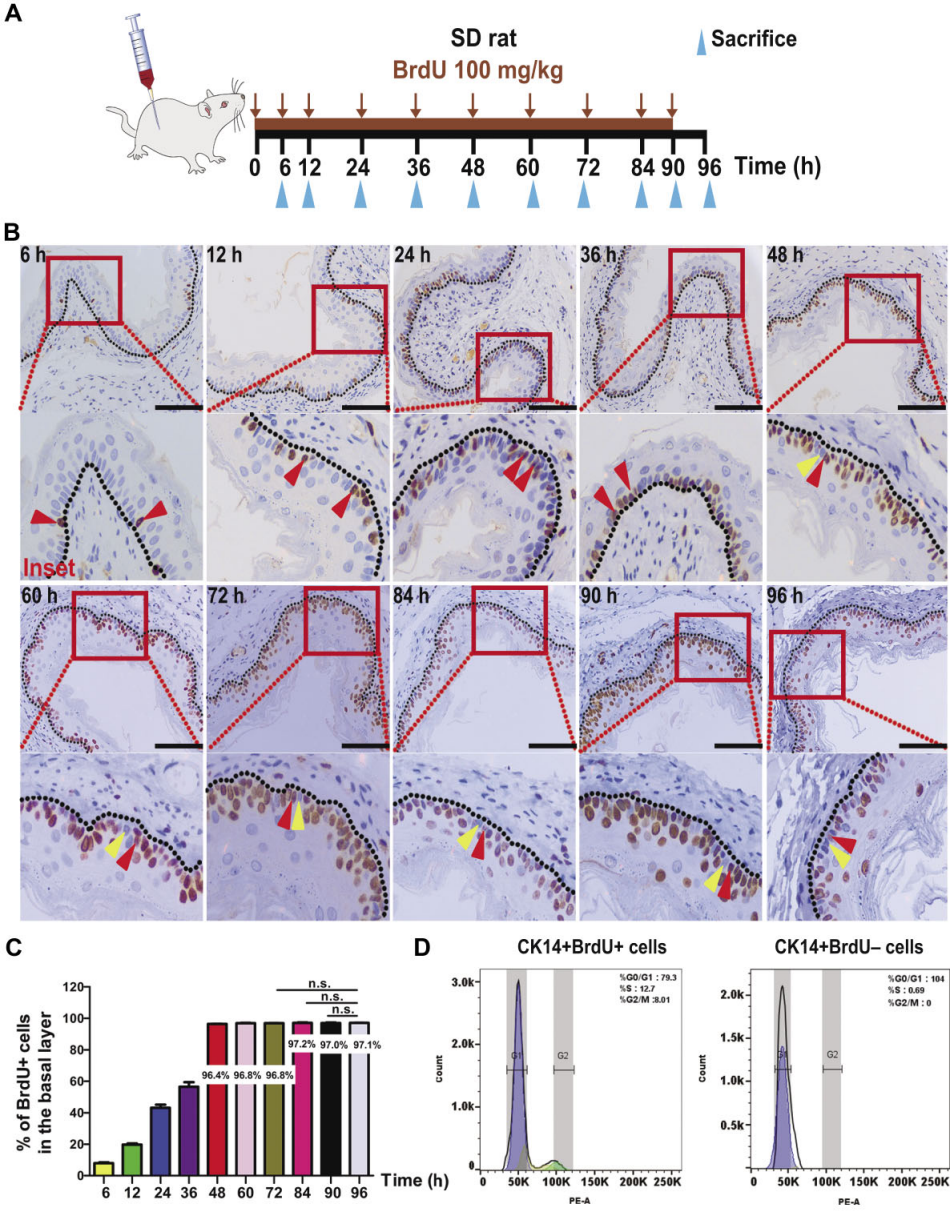
The rat esophageal basal layer has a small, relatively slow-cycling/quiescent cell population. (**A**) Schematic illustration of the BrdU labelling experiment. SD rats were injected with BrdU at 100 mg/kg body weight once every 6 h for 4 days and sacrificed at the designated time points. (**B**) Immunohistochemical staining of BrdU in esophageal sections at the designated time points. The dotted line marks the basement membrane. Red triangles indicate BrdU+ cells; yellow triangles indicate BrdU– cells. Scale bar, 100 μm. (**C**) Percentage of BrdU+ cells in the basal layer of rat esophageal epithelia (*n* = 5, each *n* represents five intact basal layers of esophageal epithelia counted at each time point). The data are presented as mean ± SD (n.s., not significant). (**D**) Cell cycle profiling of BrdU+ and BrdU– cells in the basal layers of 96 h label-chase rat esophagi by FACS.

Previous studies have shown that the stem cells in mammalian gastrointestinal tract and skin are represented by a subpopulation of relatively slow-cycling/quiescent basal cells (Sangiorgi and Capecchi, 2008; Mascré et al., 2012; Buczacki et al., 2013; Roche et al., 2015). Long-term 5-bromo-2′-deoxyuridine (BrdU) and 5-iodo-2′-deoxyuridine (IdU) label-retention experiments have indicated that a slow-cycling/quiescent basal cell population also exists in mouse and human esophageal epithelia (Okumura et al., 2003; Croagh et al., 2007). Therefore, we took a shortcut and applied *in vivo* BrdU label-chase experiments to identify the potential stem cells in rat esophageal stratified squamous epithelia. A detailed experimental flowchart is shown in Figure 1A. Sprague–Dawley (SD) or F344 rats were given 100 mg/kg BrdU by intraperitoneal injection, and BrdU-labelled rat esophageal cross-sections were obtained at the indicated time points. These sections were stained with an anti-BrdU antibody and examined by immunohistochemistry and immunofluorescence analyses (Figure 1B; Supplementary Figure S2A–C). The label-chase experiments showed that BrdU-labelled (BrdU+) cells were identified mainly in the basal layer with short labelling times whereas detected in the basal and suprabasal layers with long labelling times (>48 h). Cell counting indicated that the number of BrdU+ cells in the basal layer increased gradually and ultimately reached a maximum level at the 48 h labelling time point (Figure 1C; Supplementary Figure S2C). When BrdU labelling was performed for up to 72–96 h, the BrdU+ cells remained at the same level in the basal layer (Figure 1C; Supplementary Figure S2C). The BrdU+ and BrdU– (non-BrdU-labelled) cell percentages were 97.02% ± 0.12% and 2.98% ± 0.12%, respectively. Similar results were obtained from BrdU label-chase experiments in BALB/c mice (Supplementary Figure S2D and F).

We determined the cell cycle profiles of BrdU+ and BrdU– cells in the basal layers isolated from 96 h label-chase rat esophagi by fluorescence-activating cell sorting (FACS). The results demonstrated that in CK14-marked basal layer cells, CK14+BrdU+ cells were cycling cells, whereas CK14+BrdU– cells were slow-cycling/quiescent cells at the G0/G1 phase of the cell cycle (Figure 1D). Double staining of BrdU for potential esophageal stemness markers, namely cytokeratin15 (CK15), ITGα6, CD34, and P75NTR, and for stemness-related markers, namely SOX2, BMI1, and OCT4, revealed that the individual cell markers did not specifically and sufficiently mark the CK14+BrdU+ cycling cells or the CK14+BrdU– slow-cycling/quiescent cells in the basal layers (Supplementary Figure S3B and C), consistent with the results shown in Supplementary Figure S3A. However, we noticed that all CK14+BrdU– slow-cycling/quiescent cells in the basal layer coexpressed the stemness-related markers SOX2, BMI1, and OCT4 (Supplementary Figure S3D and E). We determined the distribution patterns of the CK14+BrdU– cells and found that they were randomly located in the basal layer. Taken together, these results indicated that the basal layer of mammalian esophagus contained a subpopulation of quiescent basal cells (QBCs). However, these cells could not be specifically or effectively marked by currently known stemness-related markers.

### Determination of QBCs as a unique cell population by genome-wide DNA methylation profiling *in vivo*

**Figure 2:**
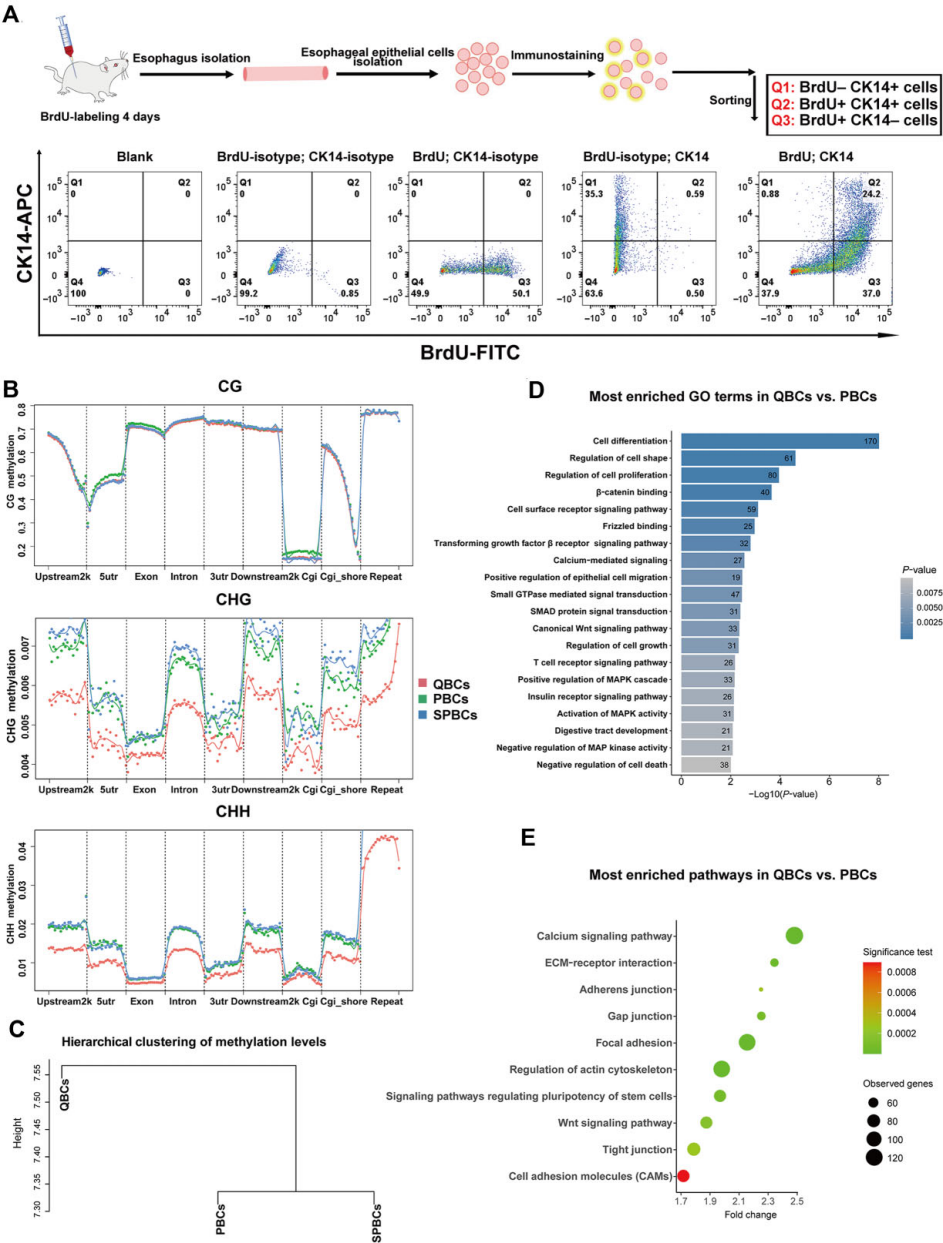
QBC population in the rat esophageal basal layer has a distinct methylation profile. (**A**) Three populations, CK14+BrdU–/QBCs, CK14+BrdU+/PBCs, and CK14–BrdU+/SPBCs, were sorted from the esophageal keratinocytes of SD rats with BrdU labelling for 96 h. (**B**) Clustering analysis of CpG, CpHpG, and CpHpH (H = A, C, and T) methylation levels among the three populations. (**C**) Hierarchical clustering in the methylation maps among the three populations. (**D**) Bar graph showing the most enriched GO terms in QBCs vs. PBCs. (**E**) Bubble plot showing the most enriched KEGG pathways in QBCs vs. PBCs.

To further define the molecular characteristics of QBCs, we decided to perform whole-genome bisulfite sequencing (WGBS) profiling experiments, as transcriptome analyses could not be performed with cells fixed and stained with a BrdU antibody. Rats were labelled with BrdU for 4 days, and then esophageal epithelial cells were sorted into three populations, CK14+BrdU–, CK14+BrdU+, and CK14–BrdU+ cells, based on BrdU and CK14 antibody staining by FACS (Figure 2A; Supplementary Figure S3A). Consistent with the immunohistochemistry and immunofluorescence results (Figure 1C), the CK14+BrdU– cells represented ∼3.50% ± 0.15% of the total CK14+ cells. We renamed the CK14+BrdU– cells QBCs, the CK14+BrdU+ cells proliferating basal cells (PBCs), and the CK14–BrdU+ cells SPBCs. WGBS profiling showed that the overall methylation levels of QBCs, PBCs, and SPBCs of *Rattus norvegicus* were similar, especially at the CpG sites, which covered > 70% of the detected methylation sites (Supplementary Figure S4A–C). However, detailed analyses of cytosine methylations at non-CpG sites, such as CpHpG and CpHpH sites (H = A, C, and T), revealed different results in QBCs, PBCs, and SPBCs. The non-CpG methylation in QBCs was significantly lower than that in PBCs and SPBCs (Figure 2B). More importantly, although the overall methylation levels of QBCs, PBCs, and SPBCs were similar, hierarchical clustering of the methylation sites based on genomic sequence clearly separated QBCs from PBCs and SPBCs, indicating that epigenetic regulation at the DNA level was distinct in QBCs compared with PBCs and SPBCs (Figure 2C).

We defined differentially methylated regions (DMRs) between QBCs and PBCs. Consistent with the results obtained from the hierarchical clustering (Figure 2C), heatmaps generated from DMR methylation levels in the top-scored gene-surrounding regions clearly displayed the differences in QBCs and PBCs (Supplementary Figure S4D and E). Gene Ontology (GO) and Kyoto Encyclopedia of Genes and Genomes (KEGG) enrichment analyses were performed on the genes with DMRs. As shown in Figure 2D, the highly enriched GO terms were mainly associated with biological processes such as cell differentiation, regulation of cell shape, and regulation of cell proliferation. The highly enriched KEGG terms were mainly associated with genes involved in focal adhesion regulation, cytoskeletal structure and regulation, adherens junctions, and the Wnt signaling pathway (Figure 2E). These results indicated that even though QBCs and PBCs were spatially close in the basal layer of the esophagus, their DNA methylation levels and patterns in the genome, especially in the gene-surrounding regions that control gene expression, were different. The epigenetic characteristics at the DNA level (DNA methylation) in QBCs were unique when compared with those in PBCs and SPBCs; thus, QBCs represented a unique cell population located spatiotemporally in the esophageal basal layer.

### Existence of QBCs in normal rat esophageal keratinocyte cell line-derived organoids

Three-dimensional (3D) organoid cultures have been demonstrated to be powerful *in vitro* systems for studying the identification, determination, self-renewal trajectory, and maintenance of tissue stem cells, as well as the formation and proliferation–differentiation homeostasis of organ tissues and carcinogenesis (Trisno et al., 2018; Whelan et al., 2018; Zhang et al., 2018; Kijima et al., 2019). To further characterize QBCs, we generated a human telomere reverse transcriptase (hTERT) immortalized rat normal esophageal keratinocyte cell line (RNE-D3; for details, see Materials and methods). Similar to rat primary esophageal epithelial keratinocytes, RNE-D3 (D3) cells in Matrigel with the conditioned culture medium formed normal and typical esophageal organoids with endodermal morphological structures, including the basal layer, the suprabasal layer, and the keratin layer, in 10–12 days (Supplementary Figure S5A–C).

Similar to the case in rat esophageal stratified squamous epithelia, an immunofluorescence assay showed that CK14, P75NTR, ITGα6, and ITGβ4 were expressed in the basal layer cells, while PCNA, BMI1, SOX2, and OCT4 were expressed in the basal and suprabasal layer cells, and CK13 was expressed in the suprabasal and keratin layer cells in D3-derived organoids (D3 organoids). These results indicated that D3 organoids could be used as a convenient *in vitro* model for our study (Supplementary Figure S5D and E).

**Figure 3:**
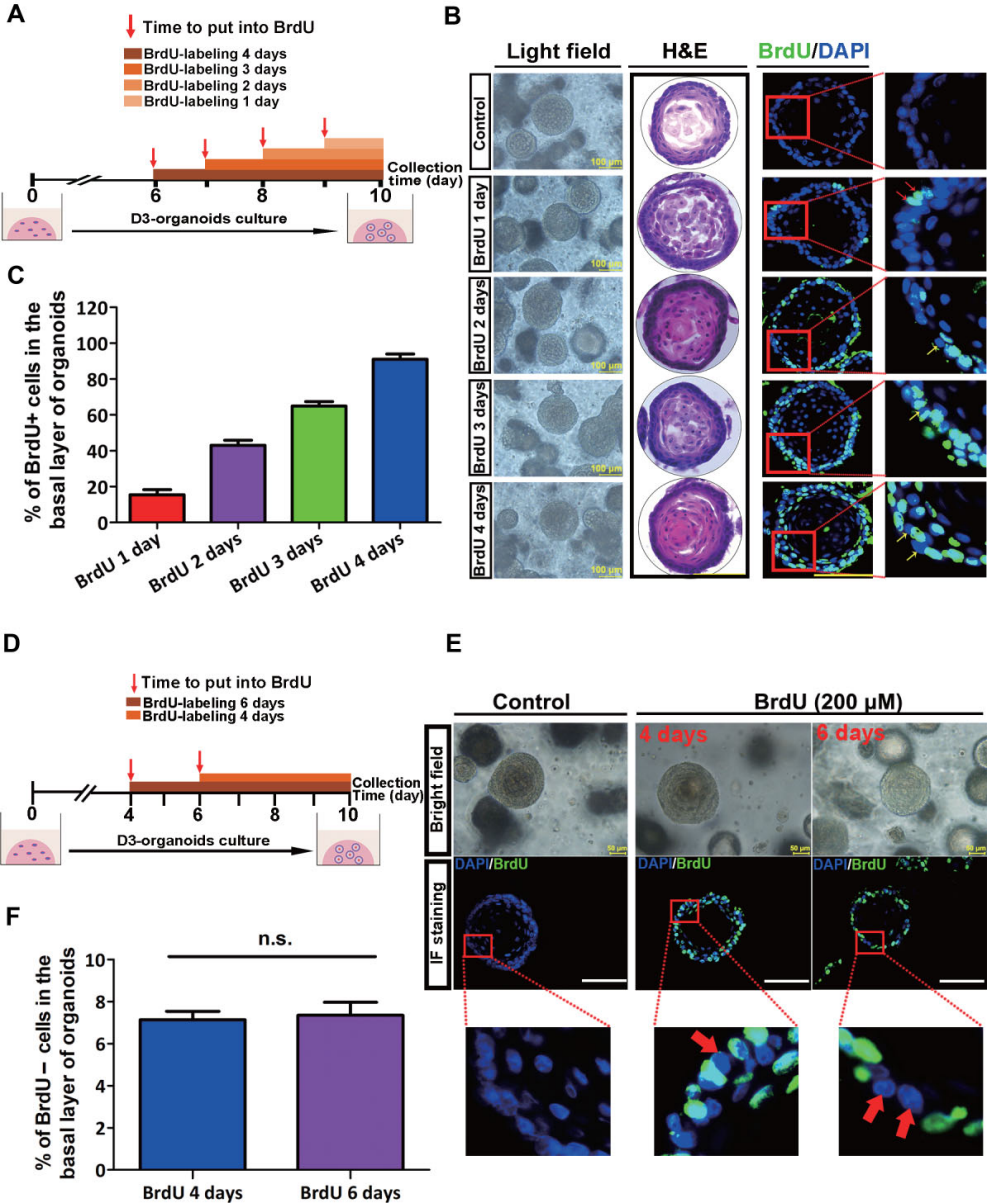
The basal layer of rat esophageal organoids derived from D3 cells contains QBCs. (**A**) Schematic illustration of the BrdU labelling experiment of rat esophageal organoids derived from D3 cells. A BrdU concentration of 200 μM was used at different time points in the organoid culture process. (**B**) Representative brightfield, H&E staining, and corresponding BrdU immunofluorescence staining images of cultured D3 organoids. Scale bar, 100 μm. (**C**) Percentage of BrdU+ cells in the basal layer of cultured D3 organoids (*n* = 9, each *n* represents nine random microscope fields, 400×). (**D**) Schematic illustration of BrdU labelling D3 organoid culture for 4 and 6 days. (**E**) Representative brightfield and BrdU immunofluorescence (IF) staining images of D3 organoids. The red arrows indicate BrdU– cells in the basal layer of D3 organoids. Scale bar, 50 μm. (**F**) Percentages of BrdU– cells in the basal layer of D3 organoids (*n* = 9, each *n* represents nine random microscope fields, 400×). The data are presented as mean ± SD for percentage analysis (n.s., not significant).

BrdU label-chase experiments were applied to determine whether QBCs were present in D3 organoids. The optimal concentration of BrdU for use in the experiments was determined to be 200 μM, as this concentration could sufficiently and effectively label cells but did not inhibit DNA synthesis in D3 organoids (Supplementary Figure S6A–D). A detailed experimental flowchart is shown in Figure 3A. Growing D3 organoids from Day 6 to Day 9 were labelled with BrdU, and then the labelled organoids were collected, fixed, and analysed at Day 10. Immunostaining showed that D3 organoids labelled with BrdU for 1, 2, 3, and 4 days displayed 15.48% ± 2.86%, 43.09% ± 2.72%, 64.97% ± 2.52%, and 93.22% ± 1.36% BrdU+ cells in the basal layer, respectively (Figure 3B and C). To ensure that the BrdU-labelled cells in the basal layers of D3 organoids reached a maximum, we prolonged BrdU labelling time in growing D3 organoids from Day 4 to Day 10 (Figure 3D). The results showed that BrdU labelling in growing D3 organoids for 6 days did not further increase the percentage of BrdU+ cells in the basal layer, compared with labelling for 4 days. The BrdU+ cells accounted for ∼92.65% ± 1.84% of the basal layer, while the BrdU– cells accounted for ∼7.35% ± 1.84% in the basal layer (Figure 3E and F). Hence, these BrdU label-chase experiments demonstrated that, similar to the rat esophageal stratified squamous epithelia, D3-derived normal esophageal organoids also contained QBCs in their basal layers.

### Resolution of QBCs at the single-cell level in rat esophageal organoids

**Figure 4:**
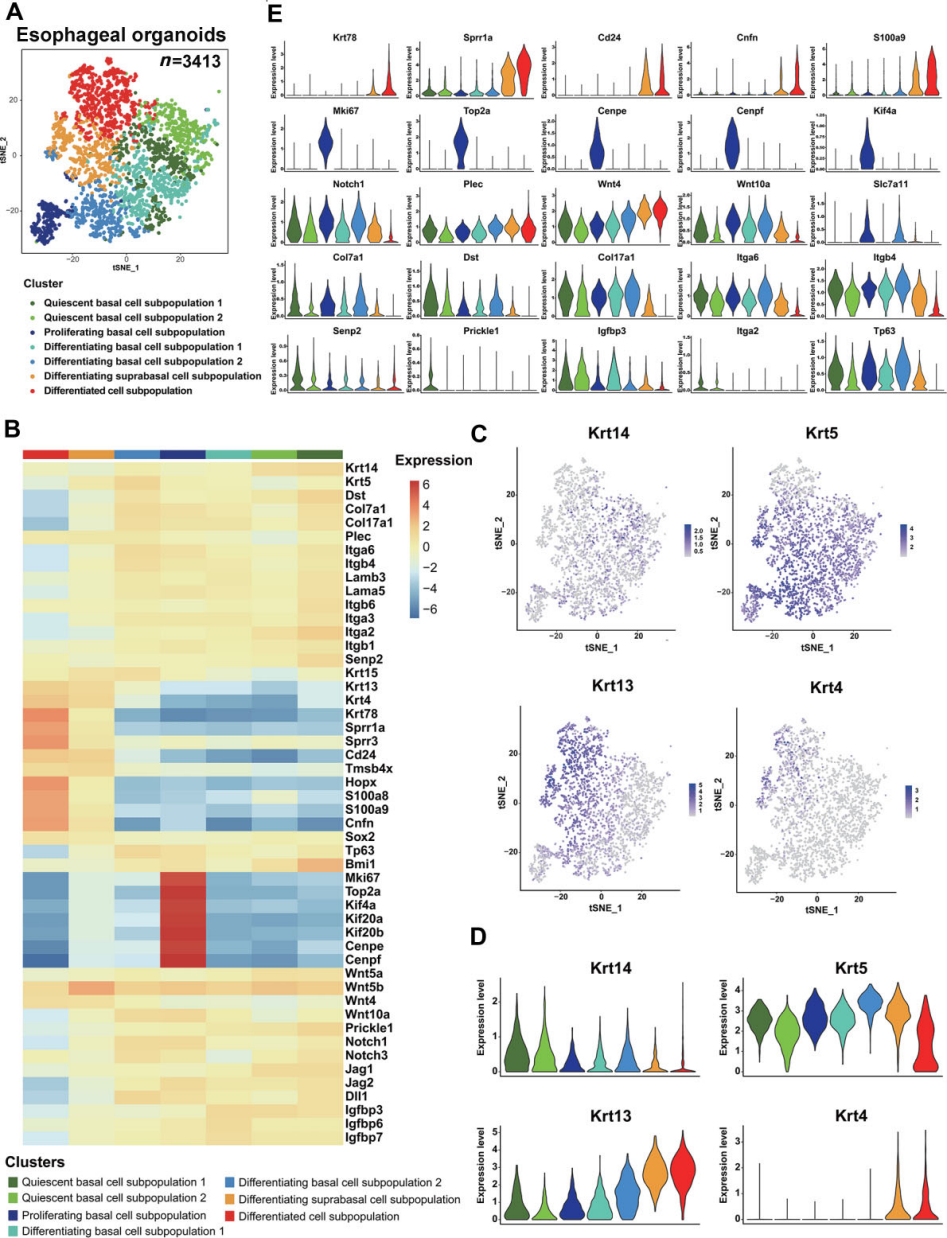
scRNA-seq analysis of rat esophageal organoids derived from D3 cells. (**A**) t-SNE plot displaying the scRNA-seq data. Different colors indicate distinct cell subpopulations. (**B**) Heatmap showing the expression of selected genes from the seven clusters corresponding to the subpopulations. (**C**) UMAP plots of keratin gene expression among the seven different subpopulations. (**D**) Violin plots of keratin gene expression among the seven different subpopulations. The y-axis represents the expression levels of the genes, and the x-axis represents the different subpopulations. (**E**) Violin plots of the expression of selected genes among the seven different subpopulations.

We performed scRNA-seq in D3 organoids to identify QBCs with distinct RNA expression patterns. D3 organoids grown for 10 days were collected, digested into single-cell suspensions, and then processed for scRNA-seq using the sorting and robot-assisted transcriptome sequencing protocol. The scRNA-seq analysis identified 3413 keratinocytes from D3 organoids that could be classified into 7 distinct but related subpopulations (Figure 4A). Based on the expression of the known keratinocyte undifferentiation-differentiation markers *CK14* (also called *Krt14*) and *CK13* (also called *Krt13*), the D3 organoid epithelial cells could be first categorized into two cell populations, *a CK14^high^* population that represented the basal cells and a *CK13^high^* population that represented the SPBCs and differentiated cells (DCs) (Figure 4B–D). The *CK13^high^* population could be further clustered into a SPBC subpopulation with high levels of *Sox2, Bmi1*, and *CK5* (also called *Krt5*) gene expression and a DC subpopulation with high levels of *Krt78, Sprr1a, Cd24*, and *Cnfn* differentiation-related gene expression (Figure 4B and E). Moreover, the *CK14^high^* population could be clustered into five subpopulations, where one subpopulation expressed high levels of cell cycle-related genes, such as *Ki67, Top2A, Kif4A, Cenpe*, and *Cenpf* (Figure 4B and E). Although the other four subpopulations all expressed low levels of cell cycle-related genes, two of the subpopulations also expressed high levels of differentiation-related gene *Notch1* and the Wnt signaling components *Wnt4 and Wnt10a* (Figure 4B and E). These two subpopulations could be further separated based on the expression levels of the proliferation-related gene *Igfbp3* (Figure 4B and E) and the reactive oxygen species (ROS)-related gene *Slc7a11* (Figure 4E). In contrast, the remaining two subpopulations expressed high levels of the negative regulators of Wnt signaling, *Senp2* and *Prickle1*, and the basement membrane markers *Col17a1* (also called *Bpag2*), *Dst* (also called *Bpag1*), *Itgα6, Itgβ4*, and *Col7a1* (Figure 4B and E). However, one subpopulation expressed these basement membrane markers at higher levels than the other.

**Figure 5:**
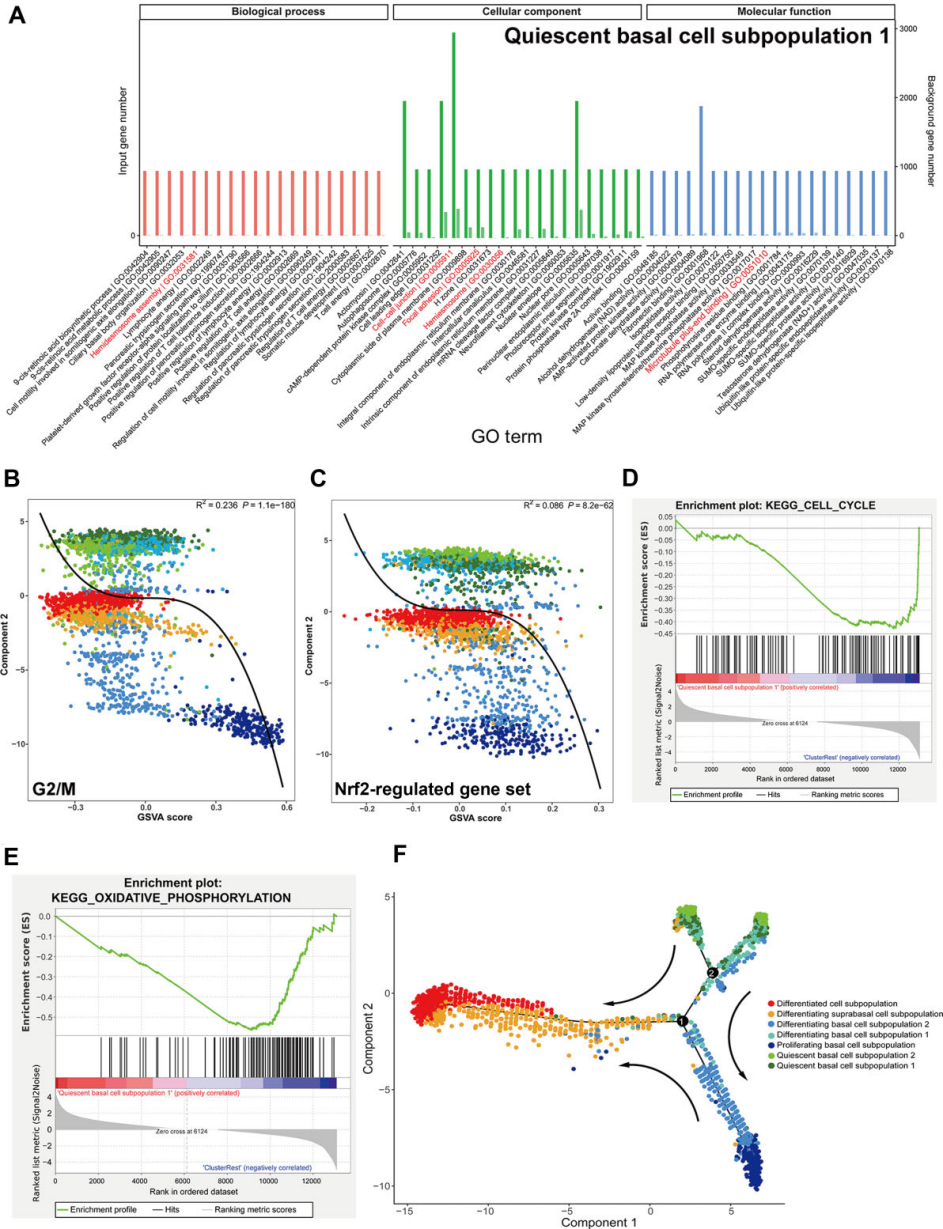
Detailed signatures of QBC1 from scRNA-seq analysis. (**A**) GO enrichment analysis of QBC1 showed significant upregulation of HD components, in accordance with related cell adhesion and cytoskeleton changes. (**B**) GSVA of genes that control cell cycle progression in the seven different subpopulations of D3 organoids. (**C**) GSVA of the Nrf2-regulated gene set in the seven different subpopulations of D3 organoids. (**D** and **E**) GSEA showed significant downregulation of cell cycle-regulating genes (**D**) and oxidative phosphorylation-regulating genes (**E**) in QBC1. (**F**) Pseudotime trajectory ordered the seven different subpopulations in a two-dimensional state-space. The x- and y-axes are two principal components. The numbers in the black circles represent nodes that determine the different cell states in the trajectory analysis. The black arrows indicate the evolution of cell fates.

Based on the results, we named seven cell subpopulations: quiescent basal cell subpopulation 1 (QBC1), quiescent basal cell subpopulation 2 (QBC2), PBC, differentiating basal cell subpopulation 1 (DBC1), differentiating basal cell subpopulation 2 (DBC2), differentiating suprabasal cell subpopulation (DSC), and DC (Figure 4). Since QBC1 and QBC2 overlapped in gene expression, we focused on QBC1/2 for further analyses, including GO analysis and gene set variation analysis (GSVA) for multiple biological functions (Hanzelmann et al., 2013; Yao et al., 2020). The results showed that the significantly enriched GO terms in QBC1/2, especially QBC1, were cell differentiation, regulation of cell shape, and terms related to the regulation of cytoskeletal structure, such as HD assembly, cell–cell junction, focal adhesion, and microtubule plus-end binding (Figure 5A).

GSVA demonstrated that QBC1/2 also displayed significantly lower expression of genes that controlled cell cycle progression, ROS, and their related phosphorylation regulation pathways than other cell subpopulations, especially PBC (Figure 5B and C). Gene set enrichment analysis (GSEA) also indicated that QBC1 had significantly downregulated expression of cell cycle- and oxidative phosphorylation-related genes (Figure 5D and E). These results were consistent with the cell cycle profiles of the QBCs isolated from rat esophagi *in vivo* by FACS (Figure 1D). The results also demonstrated that the low expression patterns of cell cycle-related genes in QBC1/2 led to the phenotype of slow-cycling/quiescent cells at the G0/G1 phase of the cell cycle, suggesting that the QBC1/2 cells identified in scRNA-seq and the QBCs identified by BrdU label-chase experiments represented the same stemness population in the basal layer of the esophagus.

The identification of seven subpopulations from D3 organoids allowed us to perform a pseudotime cell trajectory analysis and determine the evolutionary possibilities of esophageal epithelial cell fates during proliferation–differentiation homeostasis. As shown in Figure 5F, cells in QBC1/2 could be positioned as the start points with overlaps. Pseudotime cell trajectory analysis indicated that QBC1/2 cells, as the subpopulation with the most stemness in the esophageal basal layer, produced DBC1 and DBC2 cells, which progressed into DSCs, ultimately differentiating into DCs; alternatively, QBC1/2 cells generated PBCs that, in turn, progressed into DSCs and finally differentiated into DCs.

We also attempted to perform scRNA-seq with cells isolated from rat esophagi, while Busslinger et al. (2021) recently published scRNA-seq data from human esophageal tissues. The scRNA-seq data obtained from D3 organoids were similar to the scRNA-seq data from human esophageal epithelial cells (see Discussion). In summary, at the single-cell level, we identified seven cell subpopulations in rat esophageal organoids with individual mRNA expression patterns. Two subpopulations were in the suprabasal layer, whereas five subpopulations, including the most stem-like subpopulation QBC1/2, were in the basal layer. These results, together with the results obtained from human esophageal tissues (Busslinger et al., 2021), indicated that heterogeneities at the single-cell level existed in mammalian esophageal stratified squamous epithelia, especially in the basal layers.

### High levels of HD components can mark QBCs in rat esophageal epithelia and D3 organoids

Since QBC1/2 cells and QBCs represented the same stemness-exhibiting population in the basal layer of the esophagus, we compared the results obtained from scRNA-seq and WGBS, especially the genes identified by scRNA-seq in QBC1/2 and by WGBS in QBCs. The results from the two sequencing datasets showed that QBC1 was enriched with the expression of HD components (*Itgβ4, Col17a1, Dst*, and *PLEC*), the HD-anchoring extracellular matrix molecule *Lamb3*, and the Wnt pathway negative regulator *Prickle1* (Figure 4B), while hypomethylated sites were also detected in the sequences of these genes and/or the surrounding regions in QBCs (Supplementary Figure S4E). Consistent with these results, recent studies have shown that the maintenance of the Wnt pathway at low levels can facilitate the specification of the anterior foregut endoderm toward the esophageal progenitor cell lineage (Trisno et al., 2018; Zhang et al., 2018), while COL17A1 is required for skin keratinocyte stem cell maintenance (Liu et al., 2019). High expression of COL17A1 has also been identified as a marker of human esophageal quiescent stem/progenitor cells (Getachew et al., 2021). Hence, these results suggested that high levels of HD components and Wnt pathway negative regulators could serve as prominent markers of QBCs in the basal layer of the esophageal epithelium.

To define whether HD and Wnt signaling components could mark QBCs in the basal layers of rat esophagi, we examined their subcellular localizations in the basal layers of rat esophageal epithelia and D3 organoids. While the HD components ITGα6 and ITGβ4 showed strong fluorescence in the basement membranes of basal cells (Supplementary Figure S1B and D), another HD component, PLEC, and the Wnt pathway negative regulator Prickle1 were stained in both basal cells and SPBCs (Supplementary Figure S1E). In contrast, another HD component, COL17A1, displayed obvious cell staining variations in the basement membranes of basal cells (Supplementary Figure S1E).

To determine whether COL17A1 could mark QBCs, we examined the expression and subcellular localization of COL17A1 in the basal layers of rat esophagi labelled with BrdU for 4 days. High levels of COL17A1 were detected in QBCs (BrdU–) compared with PBCs (BrdU+) in esophageal epithelial tissues (Figure 6A). Similar results were also obtained in D3 organoids labelled with BrdU for 4 days (Figure 6A). Notably, high levels of DST, also a component of HDs, were detected in QBCs compared with PBCs (Supplementary Figure S8A and B). Thus, these results demonstrated that the HD components COL17A1 and DST, which were identified from the scRNA-seq data of QBC1/2, were highly expressed in QBCs, demonstrating that QBC1/2 populations identified by scRNA-seq and QBCs by WGBS were the same cell population(s) in the basal layers of the esophageal epithelia. COL17A1^high^ and DST^high^ expression could be used as prominent markers of stem cells in the basal layer of the esophageal epithelium.

**Figure 6:**
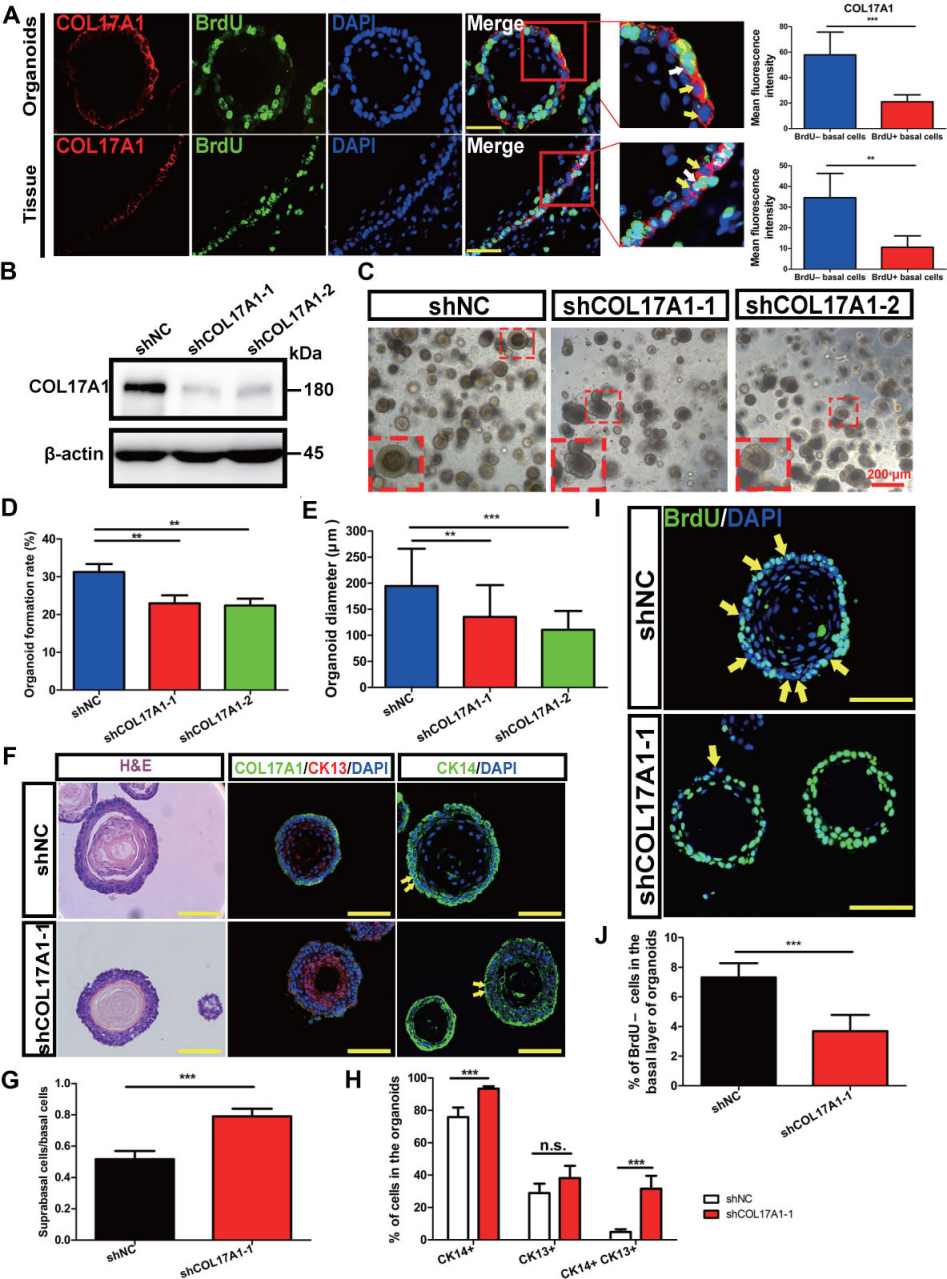
COL17A1 in stem cell maintenance and proliferation–differentiation homeostasis of rat esophagi and organoids. (**A**) COL17A1 expression was significantly higher in BrdU– basal cells than that in BrdU+ basal cells in D3 organoids and esophageal tissue. The white and yellow arrows indicate representative BrdU+ and BrdU– basal cells, respectively. The histograms display the levels of COL17A1 fluorescence intensity quantified using Fiji ImageJ. Scale bar, 50 μm. (**B**) Western blotting verification of D3-shCOL17A1 cell line construction. (**C**) Representative brightfield images of organoids at Day 10. Scale bar, 200 μm. (**D**) Quantification of the OFR. (**E**) Quantification of the

**Figure 6:**
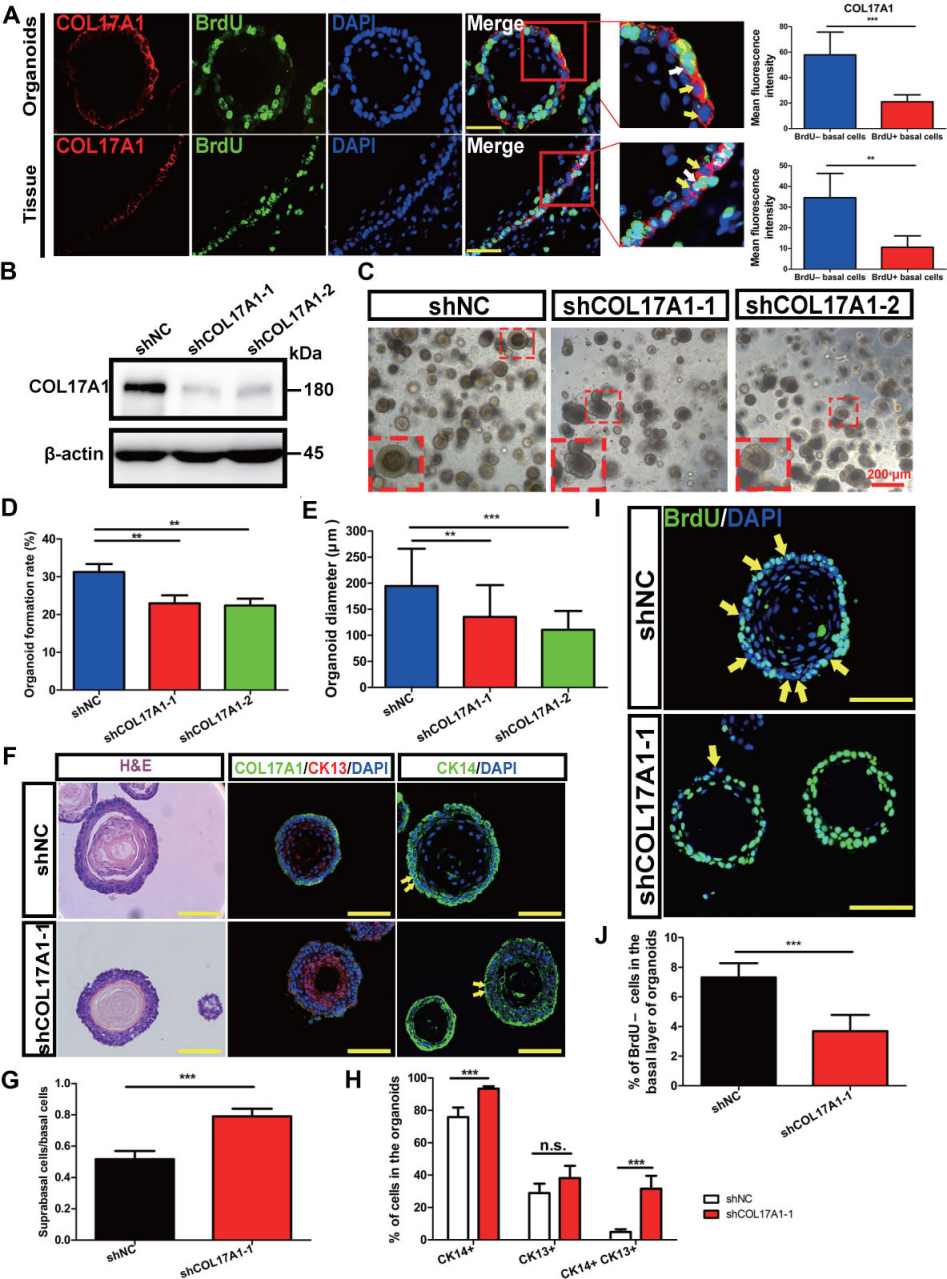
*(Continued)* organoid diameter. (**F**) H&E and immunofluorescence staining of CK13 and CK14 showed uneven basal layers and abnormal distribution of CKs after COL17A1 knockdown. (**G**) Ratio of SPBCs vs. basal cells of D3 organoids (*n* = 5, each *n* represents five random microscope fields, 400×). (**H**) Percentages of CK14+, CK13+, and CK14+CK13+ cells in D3 organoids (*n* = 5, each *n* represents five random microscope fields, 400×). (**I**) Immunofluorescence staining of BrdU in D3 organoids labelled for 4 days. The yellow arrows indicate the BrdU– cells. Scale bar, 100 μm. (**J**) Percentage of BrdU– cells in the basal layer of D3 organoids (*n* = 6, each *n* represents six random microscope fields, 200×). The data are presented as mean ± SD (**P < 0.01, ***P < 0.001).

### Roles of HDs and/or Wnt signaling in maintaining homeostasis of rat esophageal epithelia *in vitro*

To ascertain whether high levels of HD components were not only prominent markers for stem cells but also involved in regulating stem cell maintenance and proliferation–differentiation homeostasis in the basal layer of the esophagus, we suppressed HD expression via RNAi in D3 cells and examined D3 organoid formation. We first knocked down the expression of COL17A1 in D3 cells by transduction of a lentivirus expressing *Col17a1* shRNA. Immunoblotting analysis showed that lentiviral expression of *Col17a1* shRNA in D3 cells effectively and efficiently suppressed endogenous COL17A1 expression (Figure 6B). COL17A1-suppressed D3 cells and controls were grown in Matrigel with conditioned culture medium for 10–12 days. As shown in Figure 6C–E, the suppression of COL17A1 expression in D3 cells significantly affected organoid formation, e.g. it inhibited the organoid formation rate (OFR), reduced the organoid size, and perturbed the organoid shape, resulting in the destruction of proliferation–differentiation homeostasis. Immunofluorescence with anti-CK14 and anti-CK13 antibodies or immunohistochemistry with H&E staining showed that, compared with control D3 organoids, COL17A1-suppressed D3 organoids grew and developed aberrantly, forming disorganized stratified epithelia with non-smooth, rough buds, reduced sizes, and abnormal CK14 and CK13 cell staining (Figure 6F). The ratio of the suprabasal layer cells vs. the basal layer cells was increased significantly in COL17A1-suppressed D3 organoids compared with controls, indicating an imbalance in cell proliferation–differentiation homeostasis during organoid formation and growth (Figure 6G). In addition, increased numbers of CK14-positive cells were detected in COL17A1-suppressed D3 organoids, suggesting that the imbalance in homeostasis could have been caused by insufficient differentiation of these CK14-positive cells (Figure 6H). We further performed BrdU labelling experiments to measure the QBCs (BrdU–) in the basal layers of COL17A1-suppressed D3 organoids. The results showed that the percentage of QBCs in COL17A1-suppressed D3 organoids labelled with BrdU for 4 days was significantly reduced (to 3.68% ± 1.09%) in the basal layer, compared with that in control D3 organoids (7.31% ± 0.96%) (Figure 6I and J). These results indicated that COL17A1, a core component of HD and a QBC marker, was required for QBC maintenance and D3 organoid formation.

Next, we suppressed another HD component, PLEC, which is required not only for type I but also for type II HD formation (Walko et al., 2015), in D3 cells by RNAi and examined D3 organoid formation. Consistent with the results obtained from COL17A1 suppression, suppression of PLEC resulted in an inhibition of the D3 OFR, a reduction in the organoid size, and the perturbation of the organoid shape, disrupting cell proliferation–differentiation homeostasis in D3 organoids (Supplementary Figure S7C–G). The percentage of QBCs in PLEC-suppressed D3 organoids was significantly reduced to 3.00% ± 1.14% in the basal layer (Supplementary Figure S7H and I).

**Figure 7:**
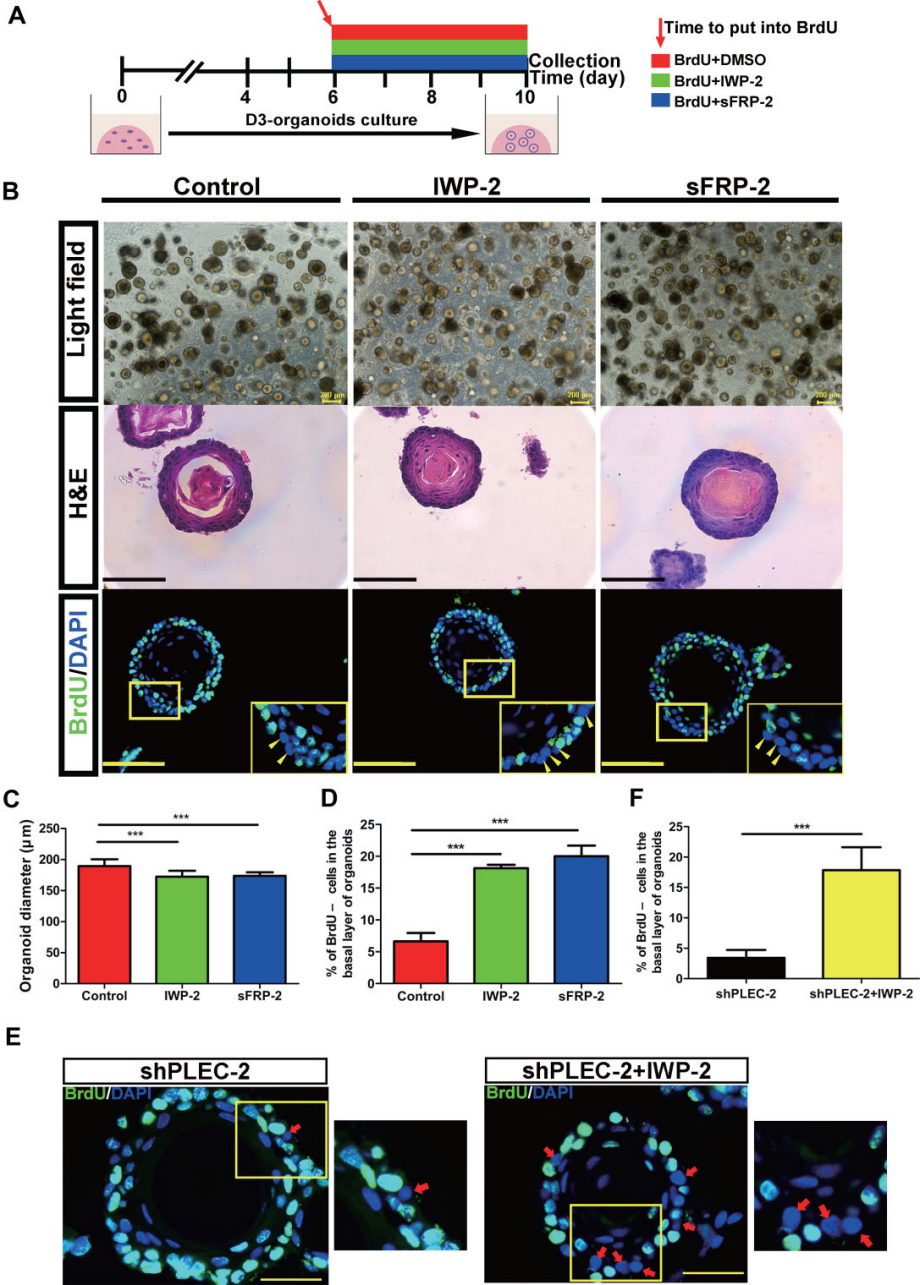
Wnt signaling–HD crosstalk maintains the stem cell identity of QBCs in esophageal organoids. (**A**) The Wnt inhibitors IWP-2 and sFRP-2 were added on Day 6 of organoid culture, and BrdU labelling experiments were performed at the same time (200 μM BrdU, 2 μM IWP-2, and 5 nM sFRP-2 were used). (**B**) Representative brightfield, H&E staining, and BrdU staining images of D3 organoids. Scale bar, 200 μm. (**C**) Quantification of the organoid diameter (*n* = 5, each *n* represents five random microscope fields, 200×). (**D**) Percentage of BrdU– cells in the basal layer of D3 organoids (*n* = 10, each *n* represents 10 random microscope fields, 400×). (**E**) Immunofluorescence staining of BrdU in D3-shPLEC organoids treated with or without the Wnt inhibitor IWP-2 for 4 days. Scale bar, 100 μm. (**F**) Percentage of BrdU– cells in the basal layer of organoids as in **E** (*n* = 6, each *n* represents six random microscope fields, 400×). The data are presented as mean ± SD (***P < 0.001).

We next determined whether Wnt signaling could also play a role in regulating QBCs, stem cell maintenance, and cell proliferation–differentiation homeostasis in the basal layers of D3 organoids. Since Wnt activities in organoids are difficult to measure directly, we performed functional assays to determine whether perturbations of Wnt activities would affect stem cell maintenance and proliferation–differentiation homeostasis in the basal cells. To this end, we perturbed Wnt activities by adding the Wnt pathway inhibitors IWP-2 (Chen et al., 2009a, b) and sFRP-2 (Galli et al., 2006; Cruciat and Niehrs, 2013) or the activator CHIR99201 (Sato et al., 2004; Sineva and Pospelov, 2010; Wray et al., 2011) to the organoid culture media and examined organoid growth and morphogenesis. A detailed experimental flowchart is shown in Figure 7A. Growing D3 organoids at Day 6 were treated with BrdU plus DMSO (control) or BrdU plus Wnt inhibitor (2 μM IWP-2, or 5 nM sFRP-2) for an additional 4 days, and then the organoid morphogenesis and the proportion of QBCs in the basal layer cells were determined by histochemistry or immunofluorescence analysis using an anti-BrdU antibody. H&E staining showed that although organoids obtained at Day 10 from either control or Wnt inhibitor treatment could differentiate into stratified squamous tissue structures with the basal layer, the suprabasal layer, and the differentiated layer, organoids treated with Wnt inhibitors were smaller in size than controls (Figure 7B and C). The BrdU label-chase experiments indicated that, compared with controls, D3 organoids treated with the Wnt inhibitors IWP-2 and sFRP-2 displayed dramatically increased numbers of QBCs in the basal layers (6.63% ± 1.31% in controls vs. 18.11% ± 0.54% or 20.01% ± 1.65% in the organoids treated with IWP-2 or sFRP-2, respectively) (Figure 7B and D). Consistently, quantitative reverse transcription–polymerase chain reaction analysis demonstrated that the mRNA expression of the Wnt pathway target genes *Axin2* and *Dvl1* in the basal cells of D3 organoids treated with Wnt inhibitors, which were isolated by FACS with an anti-ITGβ4 antibody, was significantly inhibited (Supplementary Figure S7A–C). In contrast, treatment with the Wnt pathway activator CHIR99201 induced morphologic premature differentiation of D3 organoids compared with the controls, as determined by H&E staining (Supplementary Figure S8C). Taken together, these results indicated that low levels of Wnt signaling were beneficial to the maintenance of QBCs in the basal layer, which was required for tissue homeostasis in the esophageal stratified squamous epithelium.

Finally, we explored possible crosstalk and/or interplay between HDs and Wnt signaling in the control of stem cell maintenance and proliferation–differentiation homeostasis in the esophageal stratified squamous epithelium *in vitro*. To this end, we suppressed PLEC expression in D3 organoids and simultaneously treated the organoids with the Wnt inhibitor IWP-2. Compared with D3 organoids suppressing PLEC alone, PLEC-suppressed D3 organoids treated with IWP-2 exhibited significantly rescued amounts of QBCs in the basal layers (Figure 7E and F). These results indicated that not only high levels of HDs and low levels of Wnt signaling but also crosstalk and/or interplay between HDs and Wnt signaling defined QBCs of the basal layer, which were both crucial for stem cell maintenance and proliferation–differentiation homeostasis in mammalian esophageal epithelia.

## Discussion

Despite extensive research, the identification and determination of stem cells in mammalian esophageal stratified squamous epithelia has remained controversial (Zhang et al., 2021). By genetic means in a mouse model system, Doupe et al. (2012) showed that the esophageal epithelium contains a single population of cells that divides stochastically to generate proliferating and differentiating daughter cells with equal potential, thus indicating that a ‘reserve’ slow-cycling stem cell pool does not exist in the esophageal epithelium. However, analyses of human and mouse esophageal epithelia by histology and label retention assays have demonstrated the existence of asymmetrically dividing, slow-cycling stem cells in the basal layers of the esophageal stratified squamous epithelia (Seery and Watt, 2000; Kalabis et al., 2008). Furthermore, high expression level of many stemness markers, such as SOX2, ITGα6, and ITGβ4, has been postulated to be present in the stem cells of mammalian esophageal stratified squamous epithelia (DeWard et al., 2014; Jeong et al., 2016). Although several studies suggested that high expression level of these potential stemness markers in esophageal keratinocytes promotes cell stemness, the results have often been inconclusive and controversial; thus, the topic merits further investigation (Zhang et al., 2017; Zhang et al., 2021).

In the absence of esophageal stem cell markers, long term (>3 months) tracking of DNA syntheses/cell divisions by BrdU/IdU label retention in rodent and human esophageal stratified squamous epithelia has identified a slow-cycling/quiescent stem cell population (Kalabis et al., 2008; Pan et al., 2013), which prompted us to take a shortcut by performing label-chase experiments. The BrdU label-chase experiments presented in this study allowed us to quickly and easily demonstrate that the esophageal epithelia in rats and mice as well as the D3 organoids contain a slow-cycling/quiescent basal stem cell population that accounts for ∼4%–7% of total basal cells in the basal layers. These QBCs/stem cells are spatially and randomly located in the esophageal epithelial basal layer.

Isolation of the labelled (BrdU+) and unlabelled (BrdU–) subpopulations from these label-chase experiments in rats *in vivo* with omic analyses demonstrated that hierarchical clustering in the methylation sites from WGBS could clearly separate QBCs from PBCs and SPBCs, indicating that although QBCs are spatially and randomly colocalized with PBCs in the basal layer, they are unique in terms of epigenetic regulation at the DNA methylation level. These data are in agreement with previous studies showing that stem cells and differentiation progeny cells have distinct epigenomic landscapes (Suelves et al., 2016). DNA methylation is an important epigenomic modification for stem cell maintenance, differentiation, and reprogramming (Suelves et al., 2016).

In contrast to recently reported scRNA-seq data from esophageal tissues containing epithelial cells and other types of cells (Madissoon et al., 2019; Busslinger et al., 2021), our scRNA-seq data from D3 organoids composed of only esophageal squamous epithelial cells enabled us to determine the squamous epithelial cell subpopulations in detail. These results together with *in vivo* DNA methylation profiling results indicated that QBCs represent stem cells with high levels of HDs and low levels of Wnt signaling in esophageal stratified squamous epithelia. Among them, QBCs express not only basal cell markers but also low levels of cell cycle markers, demonstrating that QBCs represent a group of cells with high levels of HD components (*Itgα6, Itgβ4, Col17a1, Dst*, and *Plec*) and Wnt pathway negative regulators *(Senp2 and Prickle1)* in esophageal stratified squamous epithelia. Pseudotime cell trajectory showed that QBCs produce proliferating and/or differentiating cells in the basal layer, which, in turn, progress into differentiating cells in the suprabasal layer and ultimately transform into differentiated keratinocytes in the differentiated layer. Thus, these results indicate that QBCs represent the stem cells in esophageal stratified squamous epithelia.

The HD components ITGα6 and ITGβ4 have been reported as markers of esophageal stem cells in previous studies (Croagh et al., 2007; DeWard et al., 2014; Jeong et al., 2016). It has been shown that SOX2^+^ITGβ4^hi^ITGα6^hi^CD73^low^ keratinocytes isolated from the esophagi of mice can form more and larger organoids than ITGβ4^low^ITGα6^low^ cells (DeWard et al., 2014). Using a 3D organotypic sphere culture system, cells isolated from mice with CD49f^+^ (also known as ITGα6^+^) and CD24^low^CD71^low^ expression have been shown to be enriched with esophageal stem cells that display elevated sphere-forming capacity and can give rise to differentiated SPBCs (Jeong et al., 2016). Recently, other HD components, such as COL17A1, were also identified as esophageal epithelial stem cell markers in a study, which showed that the stem cells in human epithelia expressed high levels of COL17A1 (Busslinger et al., 2021). Moreover, another study has shown that high expression of COL17A1 in mouse skin marks epidermal stem cells, in which the expression levels of COL17A1 controlled stem cell competition and orchestrated skin homeostasis and aging (Liu et al., 2019). Our results presented here are consistent with these data and further demonstrate that perturbation of the HD components COL17A1 and PLEC inhibits esophageal keratinocyte organoid formation, morphogenesis, and cell proliferation–differentiation homeostasis. Taken together, the results indicate that HDs are not only prominent markers for stem cells but also participate in regulating stem cell maintenance and tissue homeostasis in the basal layer of the esophagus.

Our results also reveal that low levels of Wnt signaling play crucial roles in stem cell maintenance and proliferation–differentiation homeostasis in mammalian esophageal epithelia. The Wnt pathway is tightly linked with stem cell maintenance and differentiation in multiple mammalian tissues (Steinhart and Angers, 2018; Taciak et al., 2018). In simplified columnar epithelial tissues such as intestinal niches, a gradient of Wnt signaling activity has been found along the colonic crypt axis, with the highest levels at the crypt bottom to maintain two major populations of stem cells, LGR5+ crypt base columnar (CBC) cells and +4 stem cells (Kabiri et al., 2014; Farin et al., 2016; Gehart and Clevers, 2019; Tian et al., 2019). The +4 stem cells, which reside at the +4 position in the niche above the CBC cells, have low levels of Wnt signaling compared with CBC cells, representing a slow-cycling/quiescent stem cell population (Montgomery et al., 2011; Munoz et al., 2012). Studies on the stem cells in hair follicles have shown that stem cells expressing Axin2 produce low levels of Wnt ligands to maintain their stemness during quiescence under physiological conditions (Lim et al., 2016). As our omic results obtained from scRNA-seq and WGBS pointed to QBCs that were enriched with high expression of Wnt signaling negative regulators, we manipulated Wnt signaling in D3 organoids and found that QBCs of the esophageal stratified squamous epithelium also required low levels of Wnt signaling for their maintenance. We explored the relationship between HDs and Wnt signaling and found that not only high levels of HDs and low levels of Wnt signaling but also their crosstalk and/or interplay defined QBCs of the basal layer, although further investigations are required to determine the precise underlying mechanism(s). As this paper was accepted, a study analysing the murine esophageal epithelia, 3D organoids, and human esophageal biopsies using scRNA-seq also demonstrated the cellular heterogeneity of esophageal epithelia in the context of homeostasis and aging (Kabir et al., 2022).

Based on these studies, we propose that the high numbers of HDs and low levels of Wnt signaling controlled, at least in part, by component/regulator expression regulation via epigenetic regulation at the DNA level (DNA methylation) and their crosstalk/interplay at the basal cells define the stem cells, which are required for self-renewal, maintenance, and proliferation–differentiation homeostasis in mammalian esophagi. Future work will focus on hypothesis testing, e.g. the QBCs that have strong colony/organoid formation ability after cell sorting will be investigated using HD/Wnt signaling components as markers, the QBCs that function as stem cells for the maintenance of tissue homeostasis will be examined by cell lineage tracing *in vitro* and *in vivo*, and the QBCs that are involved in regulating acute injury/damage repair in the esophageal stratified squamous epithelia will be explored.

## Materials and methods

### Study approval

All experiments involving animals complied with the standards approved by the ethical committee of National Cancer Center/National Clinical Research Center for Cancer/Cancer Hospital, Chinese Academy of Medical Sciences.

### RNE-D3 cell line and culture conditions

An hTERT immortalized rat normal esophageal epithelial cell line (RNE-D3, D3 for short) was established and preserved by our laboratory. In brief, D3 was generated from the primary esophageal basal keratinocytes of male Rat-F344 strains infected with pBABE-hTERT retroviruses. D3 is a clonal cell line with the normal Rat-F344 genome containing 42 chromosomes, including X and Y chromosomes, and one copy of hTERT cDNA integrated into chromosome 17 as determined by karyotype and whole-genome sequencing analyses.

D3 cells were cultured in DMEM/F12 (3:1) supplemented with 10% fetal bovine serum (Thermo Fisher Scientific), 8 ng/ml cholera toxin (CELL Technologies), 5 ng/ml insulin (CELL Technologies), 25 ng/ml hydrocortisone (CELL Technologies), 0.1 ng/ml epidermal growth factor (PeproTech), and 10 μM Y27632 (Topscience) in a humidified 37°C incubator supplemented with 5% CO_2_. Under these culture conditions, D3 cells continually grew (>100 passages) in our laboratory. Notably, D3 cells did not form colonies in soft agar or tumors in nude mice at early passages (<10 passages) or late passages (>50–100 passages). The OFR of D3 cells in 3D culture was ∼25%–30%, similar to that of primary esophageal keratinocytes.

D3-shCOL17A1 cell lines were constructed by lentiviral transduction using the following sequences: shRNA-1: 5′-GGACCTATCACAACAACATAG-3′ and shRNA-2: 5′-GCAGACACATTCTCAACTATA-3′.

### Organoid culture and *in vitro* BrdU labelling assay

The detailed methods of generating D3 organoids or D3-shCOL17A1 organoids are described in the Supplementary material. BrdU (200 μM, Sigma–Aldrich) was added on Day 6–Day 9 of organoid culture. After 10 days, organoids were collected from Matrigel by digestion with Cell Recovery Solution (Corning). Then, the organoids were fixed and embedded in OCT compound for subsequent experiments. For the Wnt inhibition experiment, the Wnt inhibitors IWP-2 (Tocris) and Srfp-2 (R&D Systems) were added to BrdU on Day 6 of D3 organoid or D3-shPLEC organoid culture. The Wnt activator CHIR-99021 (Tocris) was added at the beginning of D3 organoid culture. Organoid collection was performed on Day 5 and was followed by H&E staining.

### Animals and *in vivo* BrdU labelling

SD or F344 rats and BALB/c mice, both 4–5 weeks old, were purchased from Beijing Huafukang Bioscience Company. The animals were intraperitoneally injected with BrdU at 100 mg/kg body weight once every 6 h for 4 days and sacrificed at the designated time points. The entire esophagus was obtained, after which routine histological processing for H&E staining, immunohistochemistry, and immunofluorescence staining was performed.

### Sequencing and data analysis

The detailed methods of WGBS and scRNA-seq are described in Supplementary material. In brief, for WGBS, an Acegen Bisulfite-Seq Library Prep Kit (Acegen, Cat. no. AG0311) was applied for library construction. An Illumina HiSeq X Ten platform was used for final sequencing. For scRNA-seq, single cells were isolated from D3 organoids and captured in nanoliter droplets using a Chromium Single Cell B Chip Kit (10x Genomics, 1000074). scRNA-seq libraries were constructed using a Single Cell 3′ Library and Gel Bead Kit V3 (10x Genomics, 1000075), and sequencing was accomplished using an Illumina NovaSeq 6000 sequencer.

### Statistical analysis

Student's *t*-test and two-way analysis of variance were performed to analyze significant differences between groups, and *P* < 0.05 was considered to indi cate significance. The data are presented as mean ± standard deviation (SD). GraphPad Prism 7.0 was used for analysis.

## Supplementary Material

mjac038_Supplemental_File

## Notes

**Author contributions:** Y.Y. and G.D. contributed to experimentation, data interpretation, and manuscript writing. L.Q., H.Y., Xiaohong Yu, and L.X. contributed to experimentation and data interpretation. S.-H.L., W.J., and Xiying Yu contributed to project supervision and data interpretation. W.J. and Xiying Yu contributed to manuscript writing.
