# Supplementary material for: Identification and characterization of stem cells in mammalian esophageal stratified squamous epithelia
Source: J Mol Cell Biol. 2022 Jun 16;14(6):mjac038. doi: 10.1093/jmcb/mjac038 (PMC9669669; doi:10.1093/jmcb/mjac038)
Supplement: mjac038_Supplemental_File [file mjac038_supplemental_file.pdf]

Supplementary material

Identification and characterization of stem cells in mammalian esophageal stratified squamous epithelia

Yanan Yang[1,2], Guodong Deng[1,2], Lili Qiao[1,2], Hui Yuan[1,2], Xiaohong Yu[1,2], Lei Xu[1,2], Shih-Hsin Lu[1,2,3], Wei Jiang[1,2,3,*], and Xiying Yu[1,2,3,*]

[1] Department of Etiology and Carcinogenesis, National Cancer Center/National Clinical Research Center for Cancer/Cancer Hospital, Chinese Academy of Medical Sciences and Peking Union Medical College, Beijing 100021, China

[2] State Key Laboratory of Molecular Oncology, National Cancer Center/National Clinical Research Center for Cancer/Cancer Hospital, Chinese Academy of Medical Sciences and Peking Union Medical College, Beijing 100021, China

[3] Beijing Key Laboratory for Carcinogenesis and Cancer Prevention, National Cancer Center/National Clinical Research Center for Cancer/Cancer Hospital, Chinese Academy of Medical Sciences and Peking Union Medical College, Beijing 100021, China

* Correspondence to: Wei Jiang, E-mail: wjiang6138@cicams.ac.cn; Xiying Yu, E-mail: yuxiying@cicams.ac.cn

Supplementary Experimental procedures

Organoid culture

Similar to previously reported methods (DeWard et al., 2014; Giroux et al., 2017), D3 cells or D3-shCOL17A1 cells were trypsinized into single cells and resuspended in ice-cold Matrigel (Corning). A droplet of 50 μL of cell-Matrigel mixture was added into the center of each well in flat-bottom 24-well plates. After solidification in an incubator, 500 μL of advanced DMEM/F12 (Thermo Fisher Scientific) supplemented with 1× penicillin–streptomycin (Thermo Fisher Scientific), 1× N2 supplement (Thermo Fisher Scientific), 1× B27 supplement (Thermo Fisher Scientific), 10 mM HEPES buffer (CELL Technologies), 1× GlutaMAX™ (Thermo Fisher Scientific), 1 mM N-acetyl-L-cysteine (Sigma Aldrich), 100 ng/mL recombinant murine EGF (PeproTech), 100 ng/mL recombinant murine Noggin (PeproTech), 100 ng/mL recombinant human R-Spondin1 (R&D Systems) and 10 μM Y27632 (Topscience) was added to each well. 3D organoids were grown for 10-12 days at 37 °C in a CO_2 incubator, and the medium was changed every other day. The organoid

formation rate (OFR) was determined by calculating the average percentage of organoids formed from the cells initially seeded per well.

Immunohistochemistry

Paraffin-embedded sections (5-10 µm) were deparaffinized and hydrated. Citrate buffer solution (pH 6.0) was used for microwave antigen retrieval for 10 minutes. Endogenous peroxidase was blocked with 3% hydrogen peroxide solution for 20 minutes. The sections were subsequently blocked with 10% goat serum for 1 hour, incubated with primary antibodies at 4 °C overnight, and then incubated with HRP polymer for 1 hour at room temperature. DAB solution was used for chromogenic reaction under microscopic observation. Then, the sections were counterstained with hematoxylin and sealed with neutral balsam for microscopic observation. The primary antibodies used were against BrdU (Santa Cruz, 1:200), Cytokeratin14 (Abcam, 1:5000), Cytokeratin13 (Santa Cruz, 1:500) and PCNA (CST, 1:4000).

Immunofluorescence

The OCT compound-embedded esophageal tissue or organoids were cut into 5-10 µm sections using a cryosection system. The sections were blocked with 10% normal goat serum (containing 0.2% Triton X-100) for 1 hour and then incubated with primary antibodies against BrdU (Abcam, 1:200), BMI1 (Sigma, 1:200), OCT4 (Abcam, 1:200), SOX2 (Abcam, 1:500), Cytokeratin14 (Abcam, 1:1000), Cytokeratin13 (Santa Cruz, 1:500), PCNA (CST, 1:2400), P63 (Abcam, 1:100), Integrin6 (Santa Cruz, 1:500), Integrinβ4 (Abcam, 1:100), CD34 (Abcam, 1:100), P75 (Abcam, 1:50), DST (Affinity Biosciences, 1:200) and COL17A1 (Abcam, 1:200). The sections were counterstained with DAPI and sealed with Slowfade Diamond Antifade Mountant solution (Thermo Fisher Scientific) for microscopic observation. The image fluorescence intensity was measured with Fiji ImageJ. All the images were converted to 8-bit grayscale images for plot profile analysis.

FACS and cell cycle analysis

Epithelial cells were obtained from the rat esophagus as described previously (DeWard et al., 2014). Then, these cells were fixed with 2% paraformaldehyde and washed twice with PBS. The cells were permeabilized with PBS containing 0.1% Triton X-100 for 5 minutes on ice and washed with PBS 3 times. DNase I (TaKaRa) was added to each sample, and the samples were incubated for 30 minutes at 37 °C in the dark. After washing, the cells were stained with the primary antibodies anti-rat BrdU (Santa Cruz, 1:200) and anti-rabbit CK14

(Abcam, 1:5000) for 30 minutes at room temperature. Subsequently, the cells were incubated with goat anti-rat Alexa Fluor® 488 and donkey anti-rabbit APC (IgG H&L) for 30 min at room temperature. Then, a BD Flow Sorter was used to sort the BrdU+CK14+, BrdU-CK14+ and BrdU+CK14- cells. For the cell cycle assay, the final cell pellet was suspended in 400 μl of PBS containing a 1:1000 dilution of propidium iodide (PI) for 30 minutes at 37 °C with protection from light. Then, flow cytometry examination was performed. The obtained data were further analyzed with FlowJo software (version 10).

Western blot analysis

Cell samples were collected with cell scrapers in cold PBS, centrifuged and washed twice. Then, the cell pellets were resuspended and lysed in RIPA buffer (Beyotime) supplemented with a complete protease inhibitor cocktail (Thermo Fisher Scientific). BCA solution (Thermo Fisher Scientific) was used for protein concentration measurement. Protein samples were added to 4× loading buffer and denatured at 95 °C for 10 minutes. The prepared protein samples were loaded onto SDS–PAGE gels for electrophoresis and immunoblotting. PVDF membranes (Millipore) were used for protein transfer in a Tris-glycine system. The membranes with transferred proteins were blocked in TBST containing 5% BSA for 2 hours and then incubated with primary antibodies at 4 °C overnight and a horseradish peroxidase-conjugated secondary antibody (HRP polymer) for 2 hours at room temperature. SuperSignal ECL solution (Applygen) was used for protein expression detection. The primary antibodies used were against PLEC (Abcam, 1:1000) and β-actin (Sigma Aldrich, 1:5000), respectively.

q-PCR

For q-PCR, total RNA was extracted using TRIzol reagent (Ambion, USA) and reverse-transcribed into complementary DNA using a PrimeScript™ RT Reagent Kit (Takara, Dalian, China). Q-PCR was carried out using a SYBR Premix Ex Taq™ Perfect Real-Time System (Takara). The expression levels were normalized to that of the housekeeping gene GAPDH. The following primers were used: GAPDH_F: 5'-CATGCCGCCTGGAGAAAC-3'; GAPDH_R: 5'-CCCAGGATGCCCTTTAGT-3'; Axin2_F: 5'-GACAGCGAGTTATCCAGCGA-3'; Axin2_R: 5'-GTGGGTTCTCGGGAAGTGAG-3'; Dvl1_F: 5'-ATGAGGAGGACAACACGAGC-3'; and Dvl1_R: 5'-AAGTGGTGCCTCTCCATGTT-3.

Esophageal epithelial cell isolation

The rat esophagus was longitudinally opened, with the muscle layers stripped, and

incubated in dispase II (Thermo Fisher Scientific) for 30 minutes at 37 °C. Then, the epithelium was peeled off from the submucosa and dissociated in 0.2% trypsin (Thermo Fisher Scientific), 0.1% Collagenase I (Thermo Fisher Scientific) and 0.1% Collagenase IV (Thermo Fisher Scientific) for 45 minutes at 37 °C, with gentle vortexing every 15 minutes. Trypsinization was terminated with RPMI-1640 containing 10% FBS, and the suspension was filtered through a 40 μm cell strainer. The obtained cells were prepared for further staining and fluorescence-activated cell sorting (FACS) assays.

Whole-genome bisulfite sequencing and data analysis

Samples (BrdU+CK14+, BrdU-CK14+ and BrdU+CK14- cells) were isolated from the esophagi of rats labeled with BrdU for 4 days as described above. The extracted DNA samples were first examined for concentration and purity to exclude those with degradation or contamination. An Acegen Bisulfite-Seq Library Prep Kit (Acegen, Cat. No. AG0311) was applied for whole-genome bisulfite sequencing library construction according to the manufacturer’s instructions. In brief, 1 ng of unmethylated Lambda DNA was mixed with 1 μg of extracted genomic DNA and sonicated into approximately 200-500 bp fragments. Then, end repair, 5'-phosphorylation, 3'-dA-tailing and 5-methylcytosine-modified adapter ligation were performed. After bisulfite processing, PCR was performed for 10 cycles to amplify the DNA using Illumina 8-bp dual index primers. An Agilent 2100 Bioanalyzer and qPCR were used to analyze and qualify the libraries. An Illumina HiSeq X Ten platform with a 150×2 paired-end sequencing method was used for final sequencing.

FastQC software (version 0.11.7) was used for quality control of the raw data, and Trimmomatic software (version 0.36) was used for removal of adapters and unqualified data. The optimized data were mapped to the Rnor_6.0 *Rattus norvegicus* reference genome using BSMAP software (version 2.73). To be eligible for further analysis, the data had to comply with criteria of unique aligned reads and methylated cytosines with sequence depth coverage ≥5. Calculation of individual cytosine methylation levels was performed using the ratio of sequenced CpG methylated cytosine depth to the total CpG cytosine depth. Differentially methylated region (DMRs) were established using Metilene software (version 0.2-7) and were defined by ≥5 cytosine sites in the candidate region no more than 200 bp from the neighboring cytosine (30 bp for CHH). The average methylation level differences of CG-DMRs, CHG-DMRs and CHH-DMRs all had to be >0.1 between different populations. Finally, the regions established as final DMRs had to meet the criteria of a 2D KS-test p value <0.05 and a BH (Benjamini & Hochberg)-corrected p value < 0.05. To investigate the altered biological processes associated with DMR-related genes, Gene Ontology (GO)

enrichment was performed (Q ≤ 0.05 was considered to indicate significant enrichment). Next, annotated genes with DMRs overlapping on their gene bodies or in the 2 kb upstream and downstream were enriched for Kyoto Encyclopedia of Genes and Genomes (KEGG) functional analysis. The raw data files from WGBS are available at the GSA database (CRA006236, https://ngdc.cncb.ac.cn/gsa/browse/CRA006236).

Single-cell RNA sequencing and data processing

D3 organoids were collected from 24-well plates by digestion with Cell Recovery Solution on ice for 2 hours. The deposited organoids were disrupted with a digestion mixture (containing 1× collagenase I, 1× collagenase IV, and 1× trypsin) for 30 minutes at 37 °C. Then, the treated organoids were centrifuged and resuspended as single-cell solutions in PBS containing 0.04% BSA for further sequencing.

Resuspended single cells were embedded into single-cell gel beads on a Chromium Single Cell Controller (10× Genomics) with a Single Cell 3' Library and Gel Bead Kit V3 (10× Genomics, 1000075) and Chromium Single Cell B Chip Kit (10× Genomics, 1000074) following the manufacturer’s instructions. The wrapped beads contained individual cells, specific barcodes, unique molecular identifiers (UMIs), cell lysis solution and the mixture needed for reverse transcription. After reverse transcription, the obtained cDNA with specific barcodes and UMIs was mixed together for single-cell RNA-seq library construction using the Single Cell 3’ Library and Gel Bead Kit V3. Then, the final sequencing was performed using an Illumina NovaSeq 6000 sequencer with a sequencing depth of at least 100,000 reads per cell with a paired-end 150 bp (PE150) read strategy (performed by CapitalBio Technology, Beijing).

FastQC software (version 0.11.2) was used for quality control, and the obtained data were mapped to the Rnor_6.0 *Rattus norvegicus* reference genome using Cell Ranger software (version 4.0.0). Barcode counting, UMI counting, and cell filtering were performed to achieve a feature–barcode matrix and determine clusters using Cell Ranger software. The exclusion criteria for abnormal cells were a gene number less than 200, a gene number ranked in the top 1%, or a mitochondrial gene ratio more than 25%. After UMI normalization, principal component analysis (PCA) and ten principal components were used to perform dimension reduction by the K-means algorithm (version 0.17) and graph-based algorithm (version 0.17), respectively. Visualization was realized by t-SNE dimension reduction analysis (version 0.15). Then, enrichment analysis was performed using the top 20 marker genes of each cluster by means of KEGG and GO analyses (KOBAS software). Single-cell trajectories determined as pseudotime were built with Monocle (version 2.4.0). The WGCNA

R software package (version 1.51) was used for weighted correlation network analysis. Subclusters were generated from every defined cluster according to the above clustering results, and the expression of genes was further calculated. The relative expression levels of specific genes are presented as violin plots. Gene set enrichment analysis (GSEA) was performed with GSEA software (version 2.2.2.4), which uses predefined gene sets from the Molecular Signatures Database (MSigDB version 6.2). To further verify the accuracy of the cell cluster definition, GSVA (gene set variation analysis) scores for a given biological process (including fatty acid metabolism, G2/M cell cycle, glycolysis, oxidation phosphorylation) and the NRF2-regulated redox state were calculated in each cell cluster using GSVA software (version 1.30.0). An NRF2-regulated gene set including 469 genes was downloaded from the GSEA website (http://www.gseamsigdb.org/gsea/msigdb/genesets.jsp?letter=N). The raw data files of scRNA-seq are available from the GSA database (CRA006237, https://ngdc.cncb.ac.cn/gsa/browse/CRA006237).

Supplementary References

DeWard, A.D., Cramer, J., and Lagasse, E. (2014). Cellular heterogeneity in the mouse esophagus implicates the presence of a nonquiescent epithelial stem cell population. Cell Rep *9*, 701-711.

Giroux, V., Lento, A.A., Islam, M., et al. (2017). Long-lived keratin 15+ esophageal progenitor cells contribute to homeostasis and regeneration. Journal of Clinical Investigation *127*, 2378-2391.

Supplementary Figures and legends

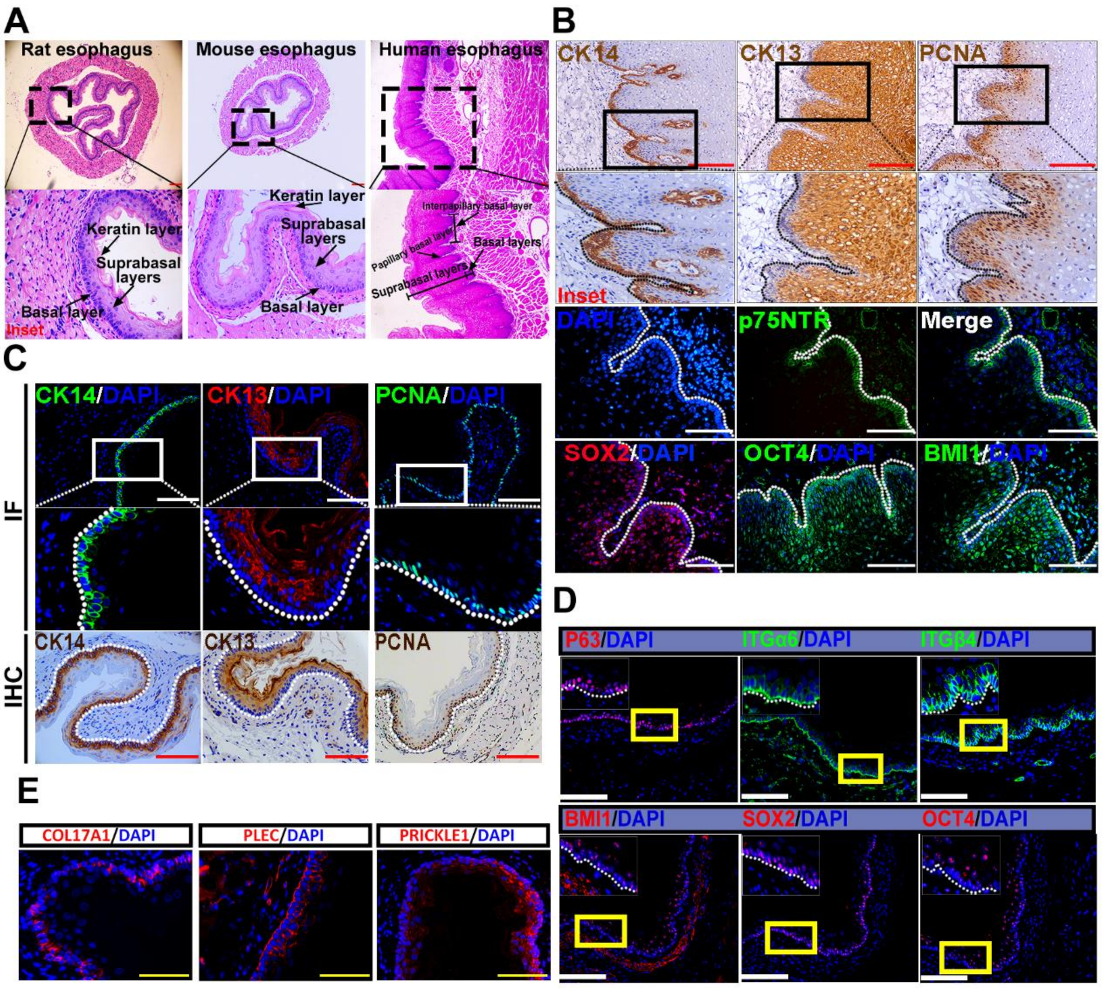

Supplementary Figure S1. Characterization of rodent and human esophageal epithelia. A H&E staining of normal rodent and human esophagus cross-sections. Rodent esophagus with endodermal structures, including the basal layer, suprabasal layers and keratin layer. Human esophagus with endodermal structures, including suprabasal layers and papillary and interpapillary basal layers. **B** Immunostaining of CK14, CK13, PCNA, P75, SOX2, OCT4 and BMI1 in human esophageal sections. **C** Immunostaining of CK14, CK13, PCNA, **D** P63, ITGα6, ITGβ4, BMI1, SOX2, OCT4 and (E) COL17A1, PLEC and PRICKLE1 in rat esophageal tissue sections. The dotted line marks the basement membrane. Scale bars: 100 μm.

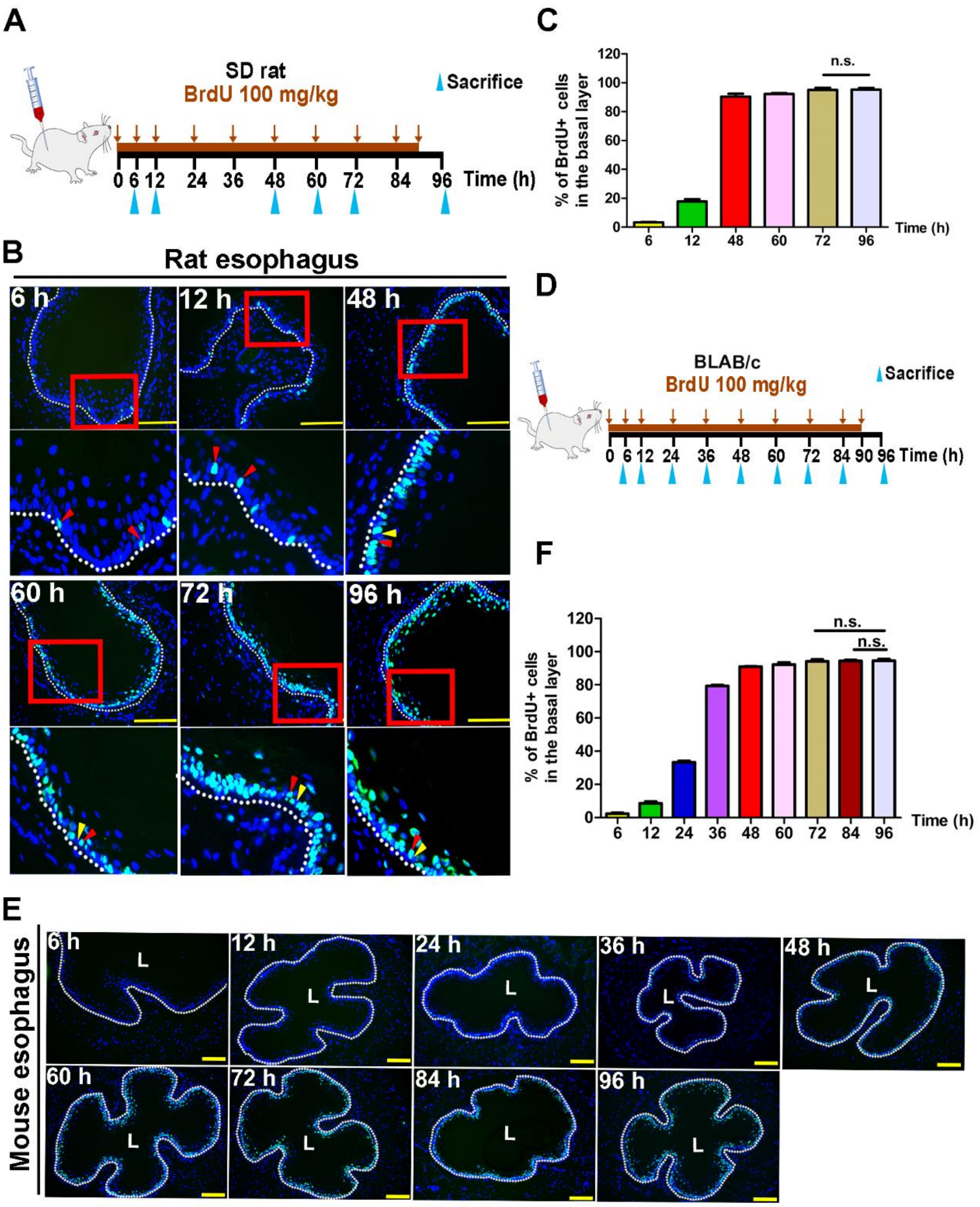

Supplementary Figure S2. The rodent esophageal basal layer exhibits a small, relatively slow-cycling/quiescent cell population. A Schematic illustration of the BrdU-labeling experiment in SD rats. SD rats were injected with BrdU at 100 mg/kg body weight once every 6 hours for 96 consecutive hours and sacrificed at five different time points. **B** Immunofluorescence staining of BrdU (green) in rat esophageal sections at the listed time points counterstained with DAPI (blue). The red arrowheads indicate BrdU+ cells; the yellow arrowheads indicate BrdU- cells. **C** Percentages of BrdU+ cells in the basal layer of the rat esophageal epithelium at the listed time points (n=5, each n represents 5 intact basal layers of esophageal epithelium counted at each time point). **D** Schematic illustration of the BrdU-labeling experiment of BALB/C mice. BALB/C mice were injected with BrdU at 100 mg/kg body weight once every 6 hours for 96 consecutive hours and sacrificed at nine different

time points. **E** Immunofluorescence staining of BrdU (green) in mouse esophageal sections at the listed time points counterstained with DAPI (blue). **F** Percentages of BrdU+ cells in the basal layer of the mouse esophageal epithelium at the listed time points (n=5, each n represents 5 intact basal layers of esophageal epithelium counted at each time point). The data are presented as the mean +/- standard deviation for percent analysis (*$p < 0.05$, **$p < 0.01$, ***$p < 0.001$). "L" indicates the lumen; the dotted line marks the basement membrane. Scale bars: 100 μm.

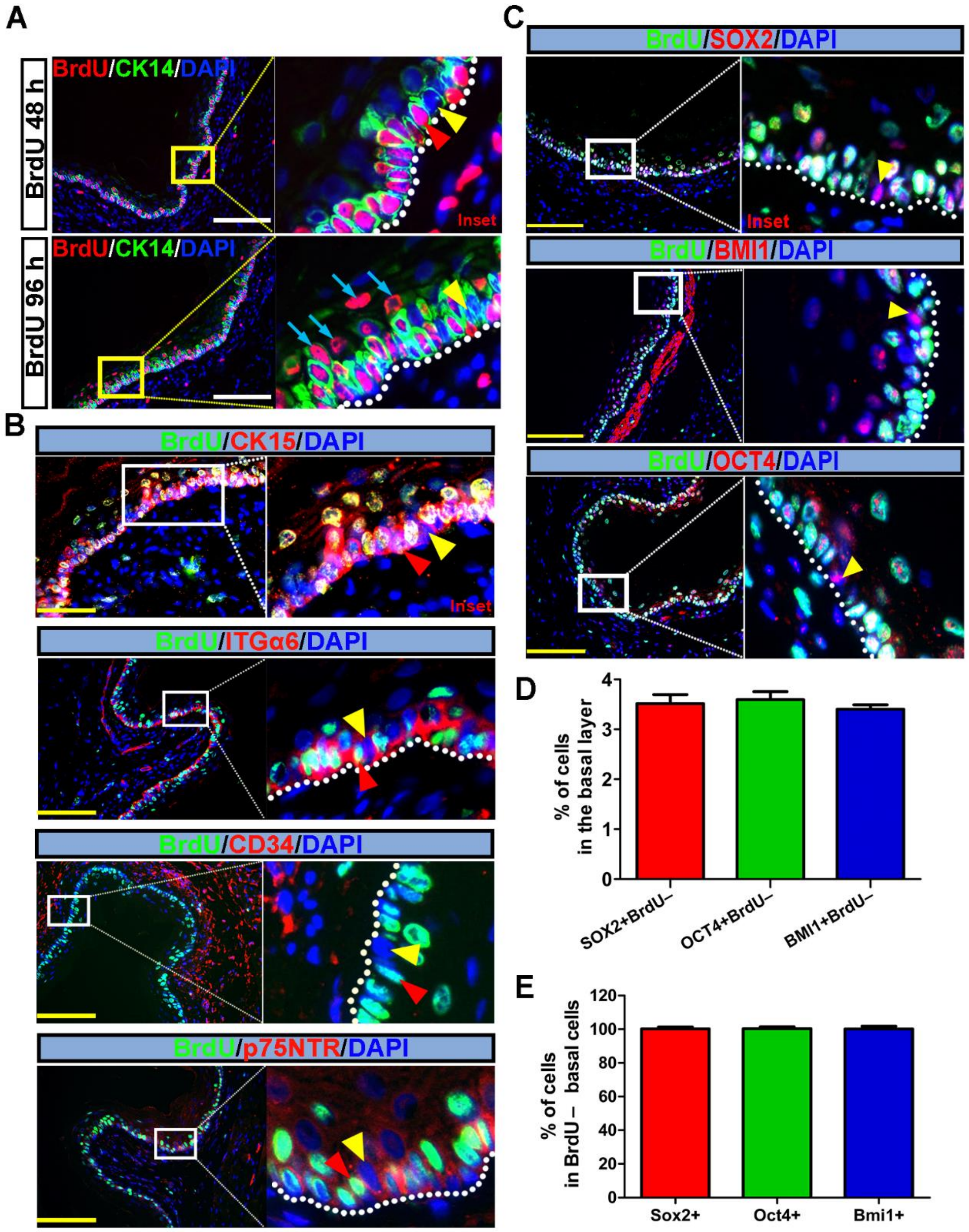

Supplementary Figure S3. Rat esophageal slow-cycling/quiescent basal cells coimmunostained for stemness markers. A CK14 (green) and BrdU (red) coimmunostaining of rat primary esophageal tissue sections counterstained with DAPI (blue). BrdU+ and BrdU- basal cells both expressed CK14 at BrdU labeling at 48 hours and 96 hours. **B** Colocalization of BrdU (green) with the potential esophageal stemness markers CK15 (red), ITGα6 (red), CD34 (red) and P75NTR (red) in rat esophageal sections at 96 h after BrdU treatment. **C** Colocalization of BrdU (green) with the stemness-related markers SOX2 (red), BMI1 (red) and OCT4 (red) in rat esophageal sections at 96 h after BrdU treatment. **D** The percentage of SOX2+BrdU- cells, BMI1+BrdU- cells and OCT4+BrdU- cells in the basal layer calculated by coimmunostaining was ~4%, which was

consistent with the percentage of BrdU- cells (n=3). **E** The percentage of SOX2+ cells, BMI1+ cells and OCT4+ cells among BrdU- basal layer cells was almost 100% as determined by manual counting (n=3). The inset panels represent magnification of regions of interest displayed with white or yellow rectangles. The yellow arrowheads indicate BrdU- cells; the red arrowheads indicate BrdU+ cells. The dotted line marks the basement membrane. Scale bars: 100 μm. The data are presented as the mean +/- standard deviation for percent analysis (*p < 0.05, **p < 0.01, ***p < 0.001).

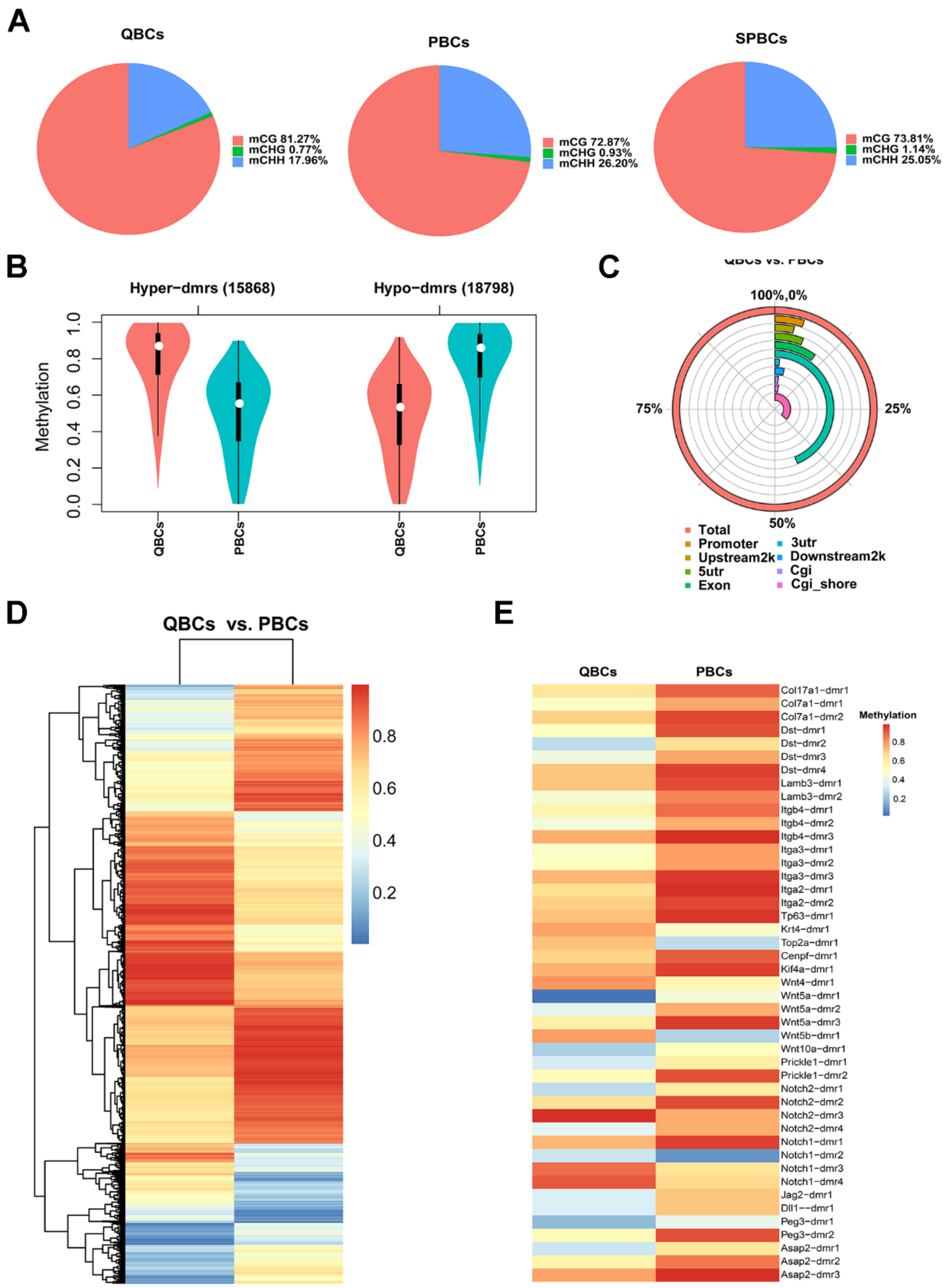

Supplementary Figure S4. Differential methylation analysis among QBCs, PBCs and SPBCs. **A** Distribution map of the methylated C sites of each population. Different colors represent methylated C sites in different contexts, and the size of each area represents the proportion of methylated C sites in the corresponding context. **B** Violin plot of the DMR average methylation level distribution between QBCs and PBCs. DMR, differentially methylated region; hyper-dmrs, hypermethylated DMRs; hypo-dmrs, hypomethylated DMRs. **C** Map of genomic functional element methylation between QBCs and PBCs. **D** Clustering heatmap of DMR methylation levels between QBCs and PBCs. **E** Heatmap of the DMR methylation levels of representative genes between QBCs and PBCs.

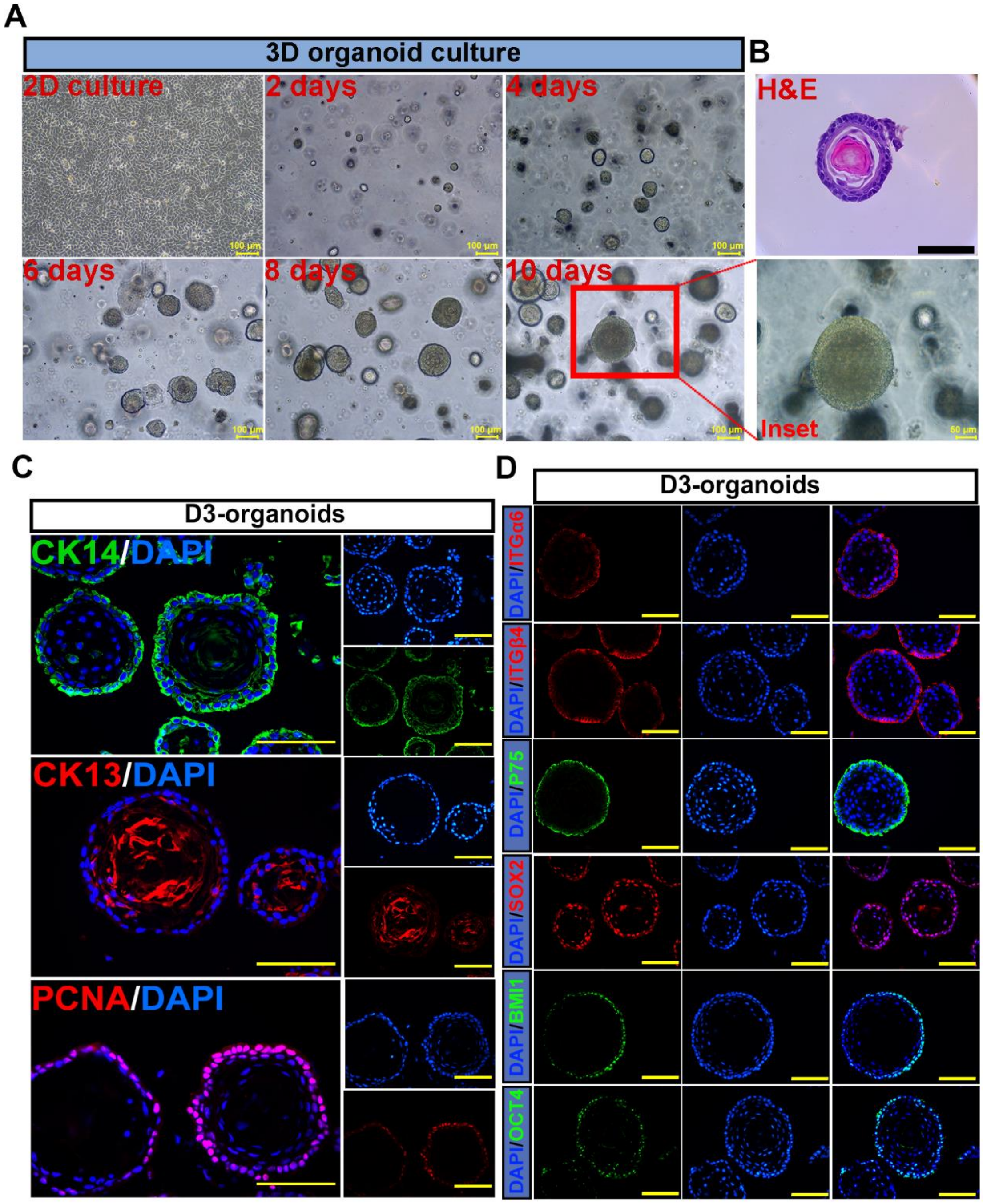

Supplementary Figure S5. Characterization of rat esophageal organoids derived from the immortalized normal rat esophageal keratinocyte cell line D3. A Expansion course of the immortalized normal rat esophageal keratinocyte cell line D3 with conditional culture to form normal and typical esophageal organoids. D3 cells in 2D culture were enzymatically dissociated and filtrated to prepare single-cell suspensions with Matrigel to initiate organoid culture in 10 days. Scale bars: 100 μm. The inset shows a representative image of a normal and typical rat esophageal organoid derived from D3 cells (D3 organoid) in a bright field. Scale bars: 50 μm. B Representative image of H&E staining of D3 organoids. Scale bars: 100 μm. **C** Immunofluorescence staining of CK14 (green), CK13 (red) and PCNA (red) with DAPI counterstaining (blue) in D3 organoids. Scale bars: 100 μm. **D** Immunofluorescence staining of the esophageal stemness markers ITGα6, ITGβ4, P75, SOX2, OCT4 and BMI1 in D3 organoids. Nuclei were counterstained with DAPI (blue). Scale bars: 100 μm.

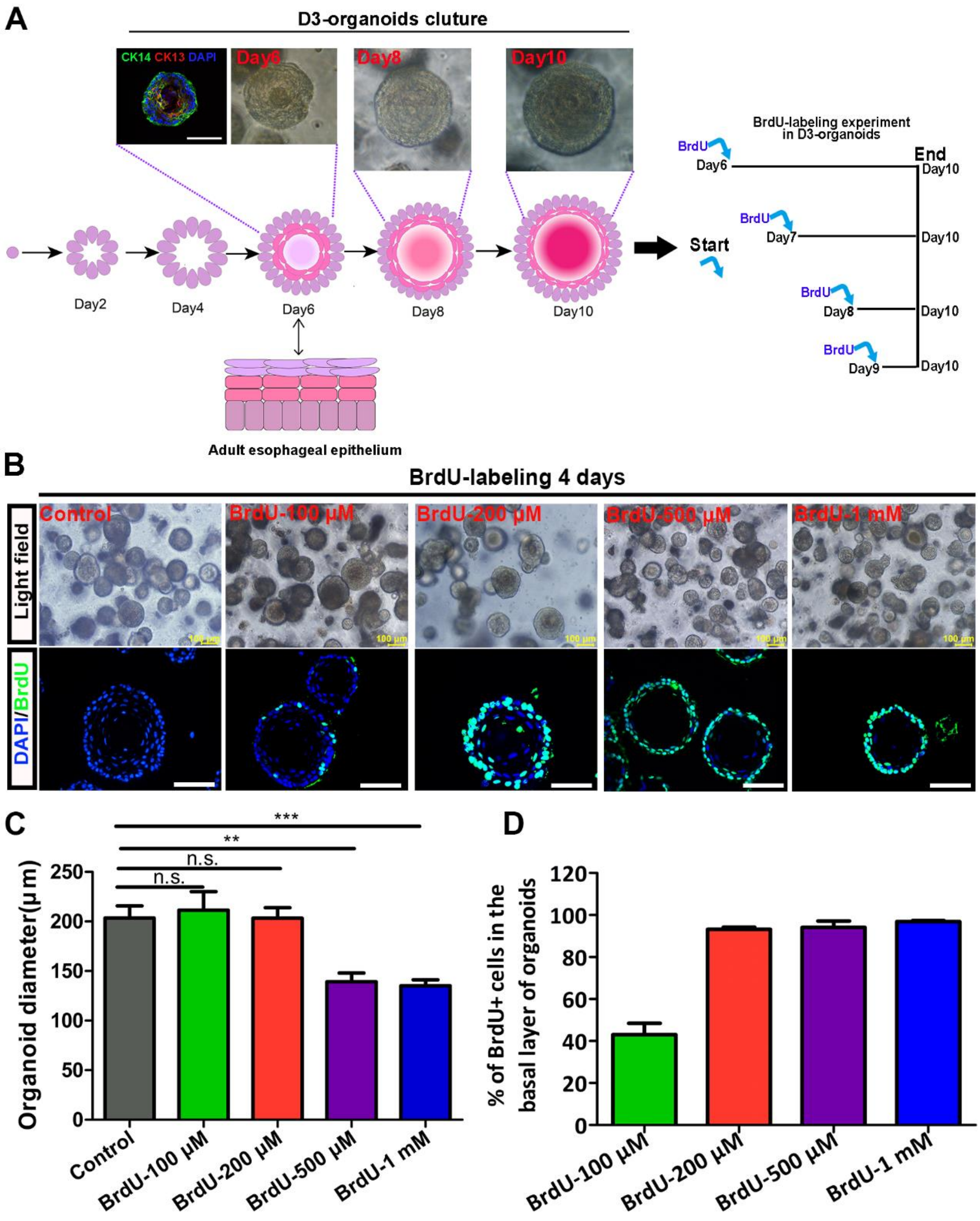

Supplementary Figure S6. BrdU labeling experiment of rat esophageal organoids derived from the immortalized normal rat esophageal keratinocyte cell line D3. A Schematic illustration of the BrdU-labeling experiment of rat esophageal organoids derived from D3. **B** The growth of D3 organoids was observed under different BrdU concentrations, and BrdU immunofluorescence staining was performed. **C** The diameters of D3 organoids were calculated under different BrdU concentrations (n=5, each n represents five random microscope fields, 200x). **D** Percentages of BrdU+ cells in the basal layers of D3 organoids under different BrdU concentrations (n=8, each n represents eight random microscope fields, 400x). Scale bars: 100 μm. The data are presented as the mean +/- standard deviation for percent analysis (*p <0.05, **p < 0.01, ***p < 0.001).

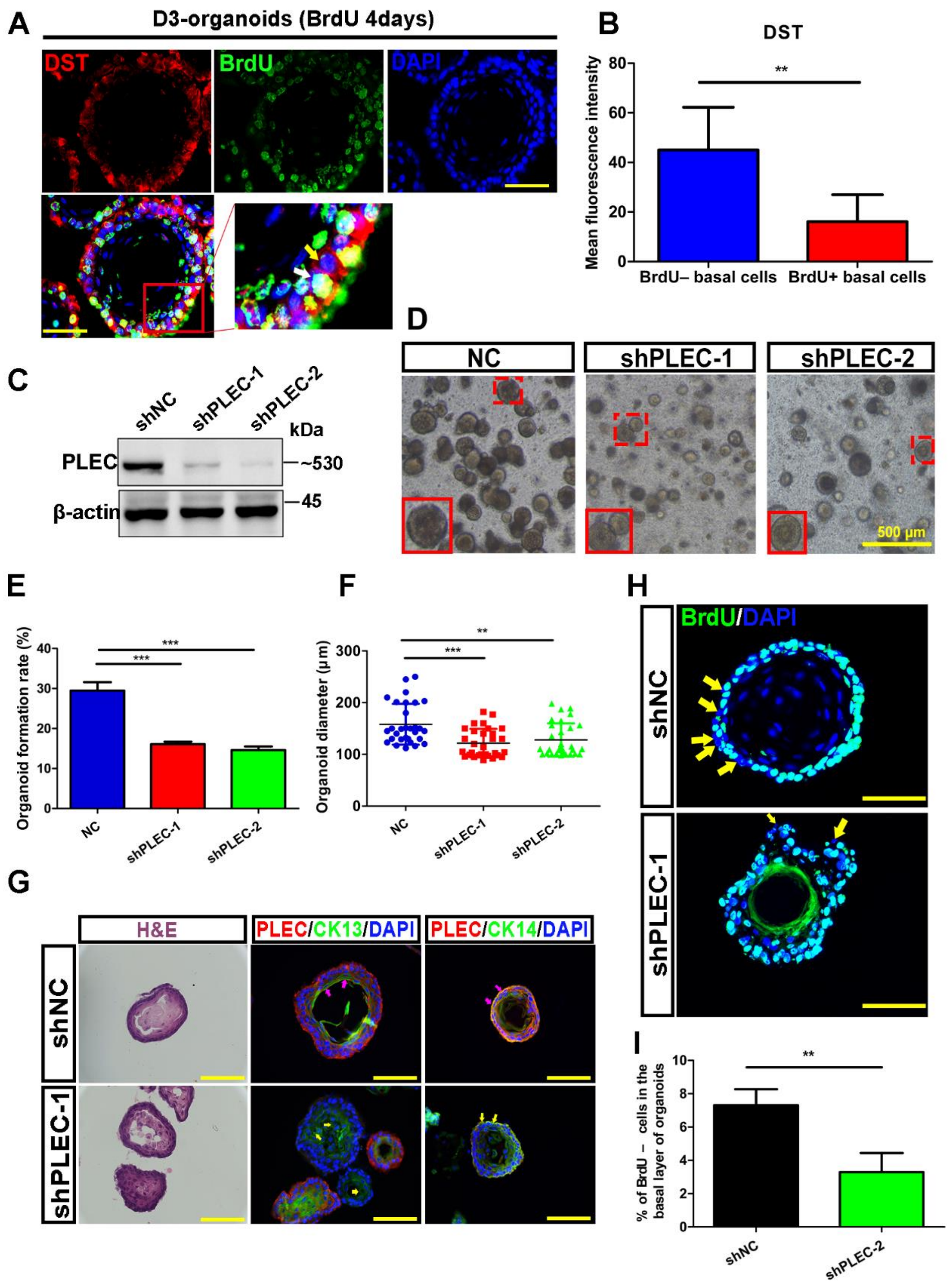

Supplementary Figure S7. Hemidesmosome (HD) components in stem cell maintenance and proliferation–differentiation homeostasis of rat esophagi and organoids. A BrdU- basal cells had higher expression of DST than BrdU+ basal cells by immunofluorescence staining in D3 organoids. Scale bars: 100 μm. **B** The mean fluorescence intensity of DST expression was quantified corresponding to **A**. **C** Western blot verification of D3-sh*PLEC* cell line construction. **D** Representative brightfield images of the organoids at Day 10. PLEC knockdown significantly inhibited organoid formation and growth. Scale bars: 500 μm. **E** Quantification of the organoid formation rate after PLEC knockdown. **F** Quantification of organoid diameter after PLEC knockdown. **G** H&E staining and immunofluorescence staining of intermediate filaments (CK13 and CK14) of

organoids showed significant self-organization perturbation presented as uneven basal layers and abnormal distribution of CKs after PLEC knockdown. **H** Immunofluorescence staining of BrdU in D3 organoids labeled for 4 days after PLEC knockdown. The yellow arrows indicate the BrdU- cells. Scale bars: 100 μm. **I** Percentages of BrdU- cells in the basal layers of D3 organoids after PLEC knockdown (n=6, each n represents six random microscope fields, 200X). The data are presented as the mean +/- standard deviation for percent analysis (*p < 0.05, **p < 0.01, ***p< 0.001).

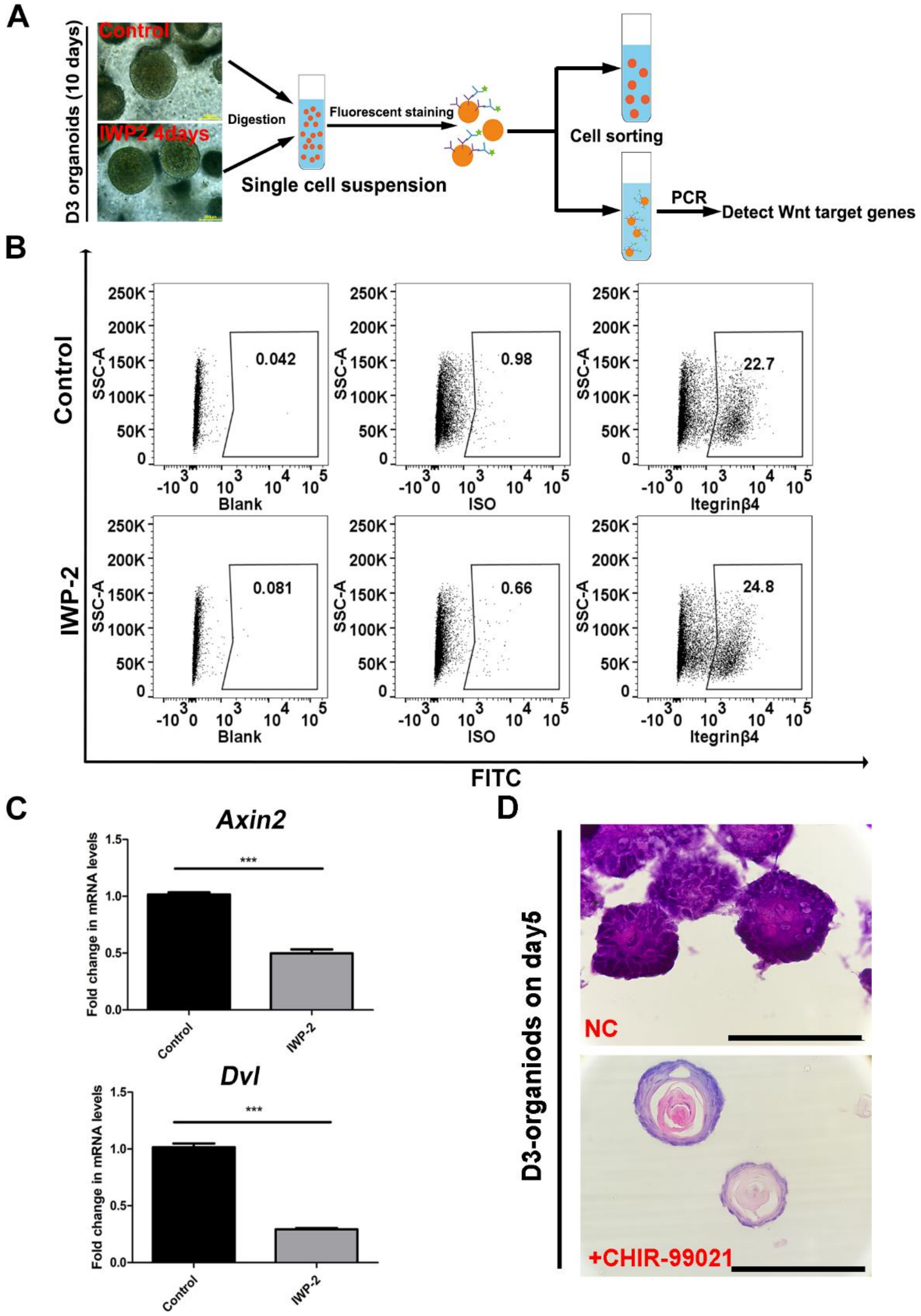

Supplementary Figure S8. Wnt inhibition in rat esophageal organoids. A Experimental procedure for obtaining basal cells from organoids Scale bars: 100 μm. **B** ITGβ4+ basal cells were sorted from D3 organoids by flow cytometry. **C** mRNA expression of Wnt downstream target genes in the group treated with the Wnt inhibitor IWP-2 and the control group. **D** The D3 organoids treated with the Wnt activator CHIR-99021 showed an increased degree of differentiation on Day 5. Scale bars: 100 μm.
